# The Tonoplastic Inositol Transporter INT1 From *Arabidopsis thaliana* Impacts Cell Elongation in a Sucrose-Dependent Way

**DOI:** 10.3389/fpls.2018.01657

**Published:** 2018-11-16

**Authors:** Sabrina Maria Strobl, Dominik Kischka, Ingo Heilmann, Grégory Mouille, Sabine Schneider

**Affiliations:** ^1^Molecular Plant Physiology, Department of Biology, Friedrich-Alexander-University Erlangen-Nuremberg, Erlangen, Germany; ^2^Department of Cellular Biochemistry, Institute for Biochemistry and Biotechnology, Martin-Luther-University Halle-Wittenberg, Halle, Germany; ^3^Institut Jean-Pierre Bourgin, INRA, AgroParisTech, CNRS, Université Paris Saclay, Versailles, France

**Keywords:** Arabidopsis, inositol, transporter, cell elongation, sucrose, seedling development

## Abstract

The tonoplastic inositol transporter INT1 is the only known transport protein in Arabidopsis that facilitates *myo*-inositol import from the vacuole into the cytoplasm. Impairment of the release of vacuolar inositol by knockout of INT1 results in a severe inhibition of cell elongation in roots as well as in etiolated hypocotyls. Importantly, a more strongly reduced cell elongation was observed when sucrose was supplied in the growth medium, and this sucrose-dependent effect can be complemented by the addition of exogenous *myo*-inositol. Comparing *int1* mutants (defective in transport) with mutants defective in *myo*-inositol biosynthesis (*mips1* mutants) revealed that the sucrose-induced inhibition in cell elongation does not just depend on inositol depletion. Secondary effects as observed for altered availability of inositol in biosynthesis mutants, as disturbed membrane turnover, alterations in PIN protein localization or alterations in inositol-derived signaling molecules could be ruled out to be responsible for impairing the cell elongation in *int1* mutants. Although the molecular mechanism remains to be elucidated, our data implicate a crucial role of INT1-transported *myo*-inositol in regulating cell elongation in a sucrose-dependent manner and underline recent reports of regulatory roles for sucrose and other carbohydrate intermediates as metabolic semaphores.

## Introduction

The cyclic sugar alcohol *myo*-inositol plays a vital role in all eukaryotic organisms and partakes in a variety of metabolic pathways. In plants, *myo*-inositol and its methylated derivatives, as, for example, pinitol and ononitol, can serve as compatible solutes to protect the plant cell from damage under salt, cold, and drought stress (Chauhan et al., 2000; Sengupta et al., 2008). Numerous phosphorylated variants of *myo*-inositol fulfill several functions in plant cells. Quite common is *myo*-inositol hexa*kis*phosphate, also known as phytate, which does not only serve as storage for phosphorus and inositol (Lott, 1984), but also acts as a co-factor for the auxin-receptor TIR1 (Tan et al., 2007) or in phosphate sensing (Wild et al., 2016). Furthermore, phytate as well as other inositol polyphosphates can serve as metabolic messengers (Valluru and Van den Ende, 2011). One of those inositol polyphosphates is inositol 1,4,5-trisphosphate (IP3), which is very well described as second messenger in the animal field, but also discussed as important second messenger in plants (Munnik and Vermeer, 2010; Munnik and Nielsen, 2011; Valluru and Van den Ende, 2011). IP3 is derived from the membrane component phosphatidylinositol-4,5-bisphosphate (PIP2). PIP2 is not only an essential membrane building block, but also plays a regulatory role among other phosphatidylinositol phosphates (Heilmann, 2016). But *myo*-inositol is not only part of cell membranes in form of phosphoinositides, it is also involved in synthesis of cell wall components. The *myo*-inositol oxygenase (MIOX) catalyzes the conversion of *myo*-inositol into glucuronic acid, which can subsequently be turned into UDP-glucuronic acid, a precursor for non-cellulosic cell wall components (Kanter et al., 2005). Evidently, inositol-containing compounds serve multiple cellular functions, and a main question has been whether and how inositol is distributed between these structural or regulatory pools.

**FIGURE 1:**
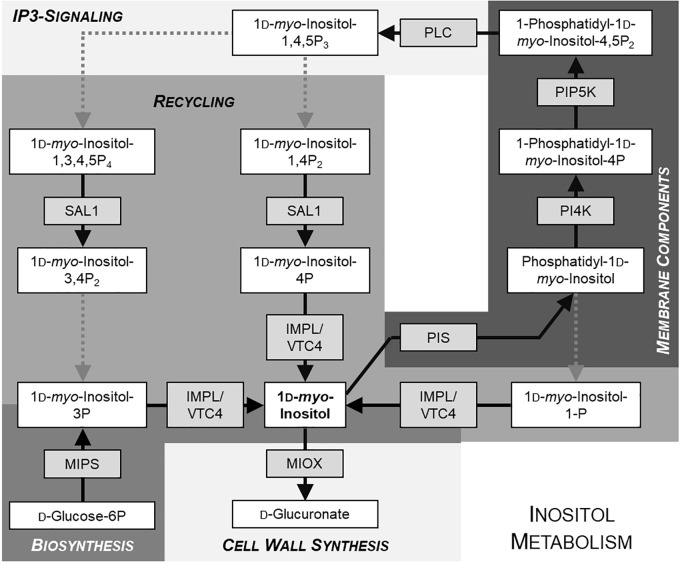
Simplified schematic overview of inositol metabolism. Metabolites are given in white boxes, the respective producing enzymes in gray boxes. Dotted arrows indicate reactions including more than one step. Different shades of background-gray highlight different pathways. Model is based on KEGG database (http://www.genome.jp/kegg/pathway.html).

*Myo*-inositol, serving as precursor for many different molecules as mentioned above, is synthesized by the conversion of glucose-6-phosphate to inositol-1-phosphate, which is subsequently dephosphorylated to *myo*-inositol. Several enzymes for the dephosphorylation of inositol-1-phosphate and other inositol-phosphates exist, for example SAL1, VTC4, and IMPL1 (Sato et al., 2011; Zhang et al., 2011; see Figure 1 for overview of inositol metabolism). The first step in *myo*-inositol biosynthesis is mediated by *myo*-inositol phosphate synthase (MIPS). In Arabidopsis, three isoforms of MIPS exist, with MIPS1 being most important (Chen and Xiong, 2010; Donahue et al., 2010; Luo et al., 2011). *Myo*-inositol is synthesized in the cytosol. Additional uptake and intracellular distribution is mediated by inositol transport proteins.

In Arabidopsis, four inositol transporter genes are present, but only three of them, *INT1*, *INT2*, and *INT4*, encode functional proteins. *INT3* was found to be a pseudogene (Schneider et al., 2007). INT1, INT2, and INT4 act as H^+^/inositol symporters and mediate import of inositol into the cytoplasm, with INT2 and INT4 being localized in the plasma membrane and INT1 in the tonoplast (Schneider et al., 2006, 2007, 2008). Targeting of INT1 to the tonoplast depends on a C-terminal acidic dileucine motif and is mediated by adaptor protein complex AP-1 (Wolfenstetter et al., 2012; Wang et al., 2014). INT1 is so far the only characterized transporter releasing *myo*-inositol out of the vacuole into the cytosol (Schneider et al., 2008; Schneider, 2015). An antagonistic actor transporting inositol into the vacuole has not been identified up to now; only a transporter for the inositol derivative phytate is known (Nagy et al., 2009). Vacuolar inositol, the substrate for INT1, is supposed to derive from phytate and/or inositol containing membrane compounds of membrane vesicles that were sequestered into the vacuole for degradation (Schneider et al., 2008; Schneider, 2015). The Arabidopsis T-DNA insertion lines with abolished (*int1.1*) or reduced (*int1.2*) INT1 activity used in this study have been characterized by our group before. While *int1.1* (SALK_085400) is a knockout line with no functional INT1 protein left, *int1.2* (SALK_018591) is a T-DNA insertion line with reduced, but residual INT1 activity (Schneider et al., 2008). Both mutant lines were shown to have shorter roots when grown on medium with sucrose, with *int1.2* seedlings displaying an intermediate root length between *int1.1* and wild type (WT) (Schneider et al., 2008). While it was postulated in the first description of *int1* mutants that the short-root phenotype might be due to inositol depletion, our most recent data presented here indicate that additional regulatory players mediate the effects of perturbed inositol distribution, such as severe length reductions in roots and also in etiolated hypocotyls of *int1* mutants. We can state that inhibition of root and hypocotyl elongation in *int1* mutants is sucrose-dependent and underlies a mechanism that can yet not be connected to any known regulatory mechanism in which inositol or its derivatives are involved.

## Materials and Methods

### Plant Lines and Growth Conditions

Used plant lines: *Arabidopsis thaliana* Columbia-0 (Lehle Seeds, Round Rock, United States), *int1.1* (SALK_085400; Schneider et al., 2008), *int1.2* (SALK_018591; Schneider et al., 2008), *mips1* (SALK_023626; Chen and Xiong, 2010; Luo et al., 2011), PIN2-GFP (Xu and Scheres, 2005).

*Int1.1* plants expressing *pPIN2:PIN2-GFP* were generated by crossing *int1.1* plants with PIN2-GFP (Xu and Scheres, 2005) plants.

WT and *int1.1* plants expressing *InsP 5-ptase* were created by introducing human InsP 5-ptase described by Perera et al. (2006) into WT or *int1.1* background via agrobacteria-mediated plant transformation (Clough and Bent, 1998) using plasmid pAF16:*35S:InsP 5-ptase*. This plant transformation vector was cloned in two steps. The *35S* promoter was amplified from plasmid pSS87 (Schneider et al., 2012) using primers e35S-5′-HindIII and e35S-3′-SbfI, verified by sequencing, and cloned via HindIII/SbfI into pAF16 (Stadler et al., 2005), yielding plasmid pAF16:*35S*. Human *InsP 5-ptase* was amplified using genomic DNA from the plant line L2-8 from Perera et al. (2006) as template with primers InsP 5-ptase-5′-SbfI and InsP 5-ptase-3′-XbaI. The obtained *InsP 5-ptase* sequence was verified by sequencing and cloned via SbfI/XbaI into pAF16:*35S*, resulting in the plant transformation vector pAF16:*35S:InsP 5-ptase*.

A complementation line was established by introducing a *pINT1::INT1* construct into *int1.1* plants. The *pINT1::INT1* construct was generated by amplifying the coding sequence of INT1 using primers AtINT1-5′SbfI and AtINT1-3′-stop-XbaI. The PCR fragment was cloned into pJET1.2 (Thermo Fisher Scientific, Waltham, United States) for verifying the sequence. The INT1 coding sequence was cloned via SbfI and XbaI in the plant transformation pSS100 (Wolfenstetter et al., 2012), which already contained the endogenous *INT1* promoter, yielding in plasmid pSSt01. *pINT1:INT1/int1.1* plants were generated by agrobacteria-mediated plant transformation (Clough and Bent, 1998).

Seeds were placed on MS-medium agar plates (Duchefa, Haarlem, Netherlands) with inositol and sucrose concentrations as indicated or on plates with 2% (w/v) of maltose, glucose, fructose, xylose, or mannitol. For all experiments, stratification was performed for 3 days at 4°C. Plates for analysis of light-grown seedlings grown under long-day conditions were then transferred to a growth chamber under a 16-h-light/8-h-dark regime at 22°C and 60% relative humidity and positioned vertically. Plates for dark-grown seedlings were exposed to light for 5 min after stratification and then kept in the dark in a growth chamber at 22°C and 60% relative humidity in an upright position. Etiolated seedlings used for RNA extraction, for determination of inositol and sugar content and for cell wall analysis were grown on a sefar-nitex membrane placed onto the MS-medium plates. Seedlings intended for ion chromatographic analysis were washed twice with sterile water before extraction.

### RNA Isolation and Quantitative Real Time-PCR

RNA was isolated from roots of light-grown seedlings or from etiolated seedlings grown on MS medium without inositol and with or without 60 mM sucrose with InviTRap^®^ Spin Plant RNA Mini Kit (Stratec, Birkenfeld, Germany). Reverse-transcription was performed using a QuantiTect reverse transcription kit (Qiagen, Hilden, Germany). For quantitative real time-PCR Brilliant III Ultra-Fast SYBR Green QPCR Master Mix (Agilent Technologies, Santa Clara, United States) and a Rotor-Gene Q real-time cycler (Qiagen, Hilden, Germany) were used. Expression levels were calculated relative to *UBQ10* (Czechowski et al., 2005). Oligonucleotides are listed in Supplementary Table 1.

### Ion Chromatographic Analysis

Inositol, fructose, and glucose content in etiolated seedlings or roots of seedlings grown under long-day conditions were analyzed as described in Schneider et al. (2008).

### Cell Wall Analysis

The analyses were performed on an alcohol-insoluble residue (AIR) of 7-day-old dark-grown hypocotyls, prepared as follows. 100 mg (FW) of ground samples were submerged into 96% ethanol and incubated for 30 min at 70°C. The pellet was then washed with 100% (v/v) ethanol, twice with acetone at room temperature. The remaining pellet of AIR was dried in a fume hood overnight at room temperature. Dry weight of samples was measured.

#### Monosaccharide Composition of the Cell Wall

After hydrolysis of AIR (10 mg) in 2M TFA for 1 h at 120°C the released monosaccharides were quantified by HPAEC-PAD as described in Harholt et al. (2006).

#### Quantification of Homogalacturonan and Methyl-Esterification Degree of the Cell Wall

After saponification of the AIR (10 mg) with 0.05 M NaOH, supernatant containing methyl ester released from the cell wall was separated from the pellet with polysaccharides. Pectins were extracted from this pellet with 0.5% ammonium oxalate at 80°C for 2 h as described (Bacic et al., 1996). Uronic acids were then quantified by colorimetry using meta-hydroxydiphenyl-sulfuric acid method as described (Blumenkrantz and Asboe-Hansen, 1973).

Methyl ester were quantified through the measurement of the methanol released during the saponification of the AIR (see above) via a colorimetric assay using enzymatic oxidation of methanol (Klavons and Bennett, 1986).

### Staining Methods, Plasmolysis, BFA Treatment, and Microscopy

Esculin and Lugol staining were performed as described in Yeats et al. (2016). Images were taken with a Zeiss Axioskop (Carl Zeiss Jena GmbH, Jena, Germany) and processed with analySIS Doku 3.2 software (Soft Imaging System, Münster, Germany). Esculin was excited with UV light (360 nm wavelength).

FM4-64 staining was performed with a 5 μM FM4-64 solution (Invitrogen, Eugene, United States) as described by Chen and Xiong (2010), except that inositol-free MS medium (with or without 60 mM sucrose) was used instead of normal MS medium. Seedlings were stained with FM4-64 for 5 min on ice, subsequently transferred to the respective MS medium (without FM4-64) and immediately (=time point 0 min) or after 15, 30, or 60 min incubation in MS medium at room temperature analyzed under the confocal laser scanning microscope. For excitation, a wavelength of 514 nm was used; fluorescence was detected between 626 and 705 nm.

MDY-64 staining was performed with a 0.25 μM MDY-64 solution (Thermo Fisher Scientific, Waltham, United States) as described by Scheuring et al. (2015), except that inositol-free MS medium (with or without 60 mM sucrose) corresponding to the growth medium was used instead of ½ MS medium for preparing the staining solution. After staining for 5 min at room temperature the seedlings were transferred to the respective MS medium where they were kept until confocal laser scanning microscope analysis.

For visualizing plasmolysis MDY-64 stained seedlings were transferred to 400 or 600 mM mannitol dissolved in MS medium without inositol and without sucrose and incubated at room temperature. After 15 min, seedlings were transferred to microscope slides and covered with the respective mannitol solution for analysis under the confocal laser scanning microscope. For MDY-64 excitation, a wavelength of 458 nm was used; fluorescence was detected between 491 and 526 nm.

Brefeldin A (BFA) treatment of *PIN2-GFP* expressing roots was performed as described by Chen and Xiong (2010), except that inositol-free MS medium (with or without 60 mM sucrose) was used instead of normal MS medium.

Propidium iodide was used in a concentration of 10 μg ml^-1^ and excited with 488 nm; emission was detected in a range between 598 and 680 nm. GFP was excited with 488 nm and detected in a range of 495–545 nm.

Images were taken with a Leica SP2 or a SP5 confocal laser scanning microscope (Leica Microsystems, Wetzlar, Germany) and processed with Leica Confocal Software 2.5 (SP2) or LAS AF (SP5) (Leica Microsystems, Wetzlar, Germany).

### Length Analyses and Statistics

For analyzing the length of root cells, 7-day-old seedlings grown under long-day conditions were treated with propidium iodide as described above. Fully elongated cells were identified by screening upward from the root tip until the cells showed no more increase in length. For measuring hypocotyl cells plants were grown in darkness for 4 days, images were taken in the elongation zone shortly above root-to-shoot-transition without further staining. ImageJ (Abramoff et al., 2004) was used for all length analyses. Basic statistics (mean values, standard deviations, and standard errors) were performed using Microsoft Excel. Differences between genotypes were analyzed using one-way ANOVA with *post hoc* Tukey HSD test.

### Extraction and Quantification of Phosphoinositide Levels

Phosphoinositides were extracted and analyzed as described previously (König et al., 2008), with the exception that fatty acid methyl esters were analyzed using a 30 m × 250 μm DB-23 capillary column (Agilent Technologies, Santa Clara, United States) and a GCMS-QP2010S-EI GC/MS-system (Shimadzu, Kyoto, Japan).

### Accession Numbers

*INT1*: At2g43330; *IMPL1*: At1g31190; *MIPS1*: At4g39800; *MIOX2*: At2g19800; *PI4Kα*: At1g49340; *PI4K*β*1*: At5g64070; *PI4K*β*2*: At5g0935; *PIN2*: At5g57090; *PIP5K2*: At1g77740; *PIP5K9*: At3g09920; *PLC2*: At3g08510; *SAL1*: At5g63980; *PIS1*: At1g68000; *PIS2*: At4g38570; *UBQ10*: At4g05320; *VTC4*: At3g02870.

## Results

### Sucrose Triggers Inhibition of Hypocotyl and Root Elongation in *int1.1* Mutants

**FIGURE 2:**
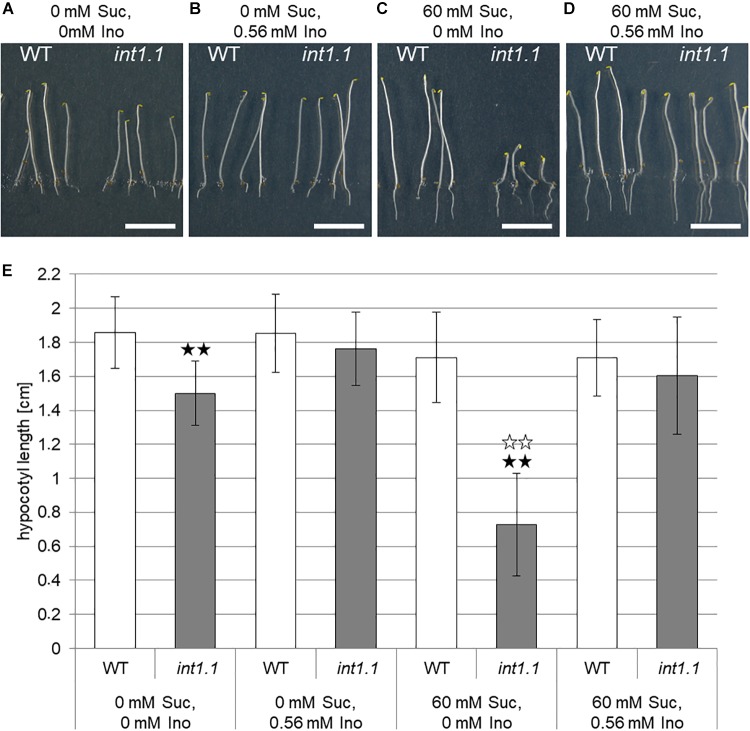
Hypocotyl length of etiolated WT and *int1.1* seedlings on media with and without sucrose and/or inositol. **(A–D)** Seven-day-old seedlings of WT and *int1.1* plants grown in the dark on sucrose and inositol concentrations as indicated. Bars represent 1 cm. **(E)** Mean values of hypocotyl lengths of etiolated WT and *int1.1* plants on different sucrose and inositol concentrations. Black asterisks indicate a highly significant difference compared to WT on the respective medium (two asterisks = *p* < 0.01). White asterisks indicate a highly significant difference between seedlings grown on medium with 0 mM sucrose, 0 mM inositol compared to the respective line grown on medium with 60 mM sucrose, 0 mM inositol (two asterisks = *p* < 0.01). Differences between genotypes were analyzed using one-way ANOVA followed by Tukey HSD test. Number of hypocotyls per line and per medium: *n* = 32–33, error bars = standard deviation.

**FIGURE 3:**
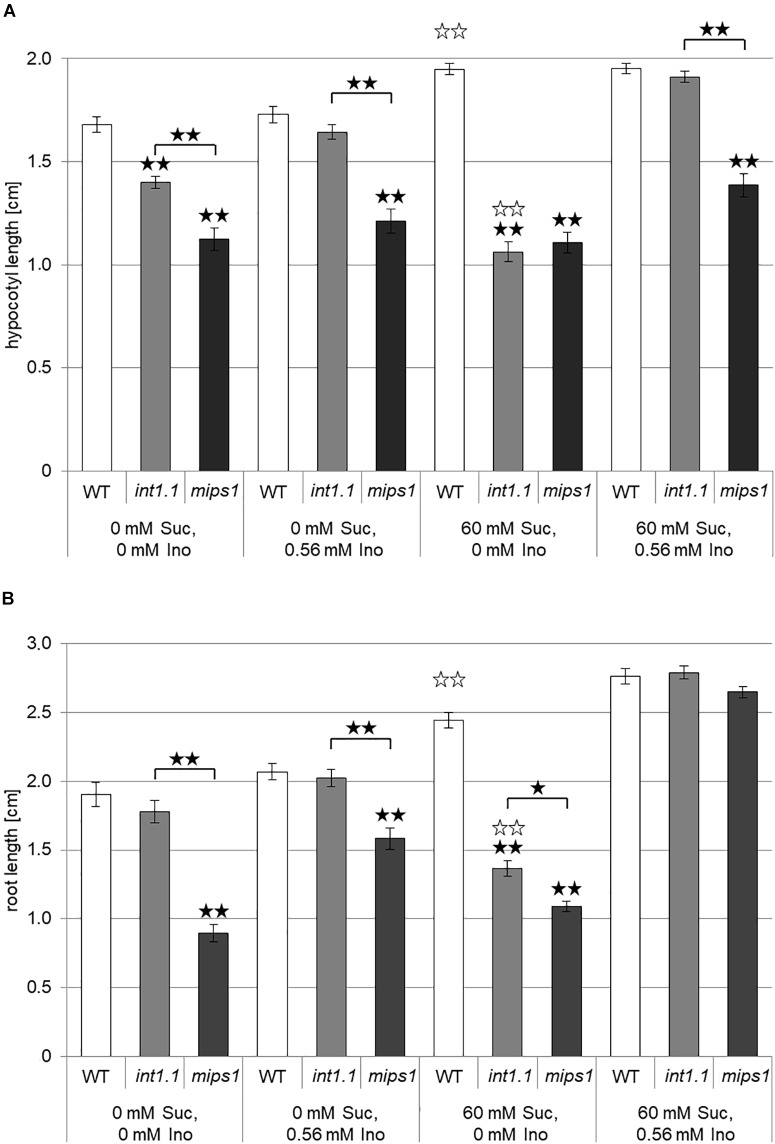
Hypocotyl length of etiolated seedlings and root length of light-grown seedlings of WT, inositol transport (*int1.1)* and inositol biosynthesis (*mips1*) mutants. Black asterisks indicate a highly significant difference compared to WT on the respective medium or between two different lines when marked with brackets (one asterisk = *p* < 0.05, two asterisks = *p* < 0.01); white asterisks indicate a highly significant difference between lines on medium with 0 mM sucrose, 0 mM inositol and the same plant line on medium with 60 mM sucrose, 0 mM inositol (two asterisks = *p* < 0.01). Differences between genotypes were analyzed using one-way ANOVA followed by Tukey HSD test. **(A)** Mean values of hypocotyl lengths of 7-day-old etiolated WT, *int1.1* and *mips1* seedlings on different sucrose and inositol concentrations. Number of hypocotyls per line and per medium: *n* = 86–100, error bars = standard error. **(B)** Statistical analysis of root lengths of 7-day-old WT, *int1.1* and *mips1* seedlings grown on different sucrose and inositol concentrations under long-day conditions. Number of roots per line and per medium: *n* = 39–40, error bars = standard error.

In our previous work, we analyzed an *INT1* knockout (*int1.1*) T-DNA insertion line for phenotypic differences compared to WT plants (Schneider et al., 2008). We examined root growth on MS medium with 2% sucrose (=60 mM) as carbohydrate supply and observed a reduced root length of *int1.1* plants grown under normal long-day conditions (16 h light/8 h dark). This length reduction could be complemented by 100 mg/l (=0.56 mM) *myo*-inositol (Schneider et al., 2008). While at first approximation, these results appeared satisfactory to explain the root phenotypes, additional experiments have been performed since which indicate a more intricate web of regulatory events. In further experiments, we included analysis of dark-grown hypocotyls as organs that underlie an extensive cell elongation in addition to the analysis of roots (Figure 2). In these experiments, *int1.1* plants grown in the dark on MS medium containing 2% sucrose surprisingly displayed severely shorter hypocotyls compared to WT (Figures 2C,E), giving similar results as light-grown roots. As we expected a more severe effect on length reduction without any carbohydrate supply, etiolation of *int1.1* and WT seedlings was carried along on MS medium without any sugar. In fact, we could observe a highly significant (*p* < 0.01) length reduction in 7-day-old dark grown *int1.1* hypocotyls on both media (Figures 2A,C,E). This length reduction could be complemented by addition of 0.56 mM *myo*-inositol (Figures 2B,D,E). But, surprisingly, by comparing the extent of length reduction of etiolated *int1.1* hypocotyls on the different growth media, we observed that the hypocotyl elongation was severely more impaired on medium supplemented with 60 mM sucrose than on medium without sugar (Figure 2E, indicated by white asterisks; *p* < 0.01). To analyze if this sucrose-triggered effect also occurs in roots we compared root lengths of WT and *int1.1* plants on media with and without sucrose. A similar situation as for etiolated hypocotyls was found: *int1.1* seedlings grown on plates without sucrose had about the same root lengths as WT seedlings. In contrast, sucrose supply resulted in a highly significant length reduction of *int1.1* roots – not only compared to WT on 60 mM sucrose, but also when compared to *int1.1* on 0 mM sucrose (Figure 3B). Thus, addition of sucrose to the growth medium resulted in a severe inhibition of *int1.1* hypocotyl and root elongation.

To examine if this effect is due to osmotic stress and if other carbohydrates have the same effect as sucrose, lengths of etiolated hypocotyls or roots of seedlings grown under long-day conditions were analyzed after growth on 2% mannitol, maltose, glucose, fructose, or xylose (Supplementary Figure 1). Mannitol addition did not alter the hypocotyl or root length of *int1.1* mutant seedlings compared to WT, excluding an osmotic effect causing the reduced hypocotyl and root elongation in *int1.1* plants. Growth on medium containing different sugars resulted in some (maltose, glucose, and fructose) or no (xylose) length reduction of *int1.1* etiolated hypocotyls or roots of seedlings grown in the light. In contrast to the strong effect of sucrose, which gave rise to a length reduction of about 50% in *int1.1* hypocotyls and roots, maltose, glucose, and fructose had distinctly less impact. Moreover, the effects of maltose, glucose, and fructose on elongation varied between hypocotyls and roots, while sucrose impaired both hypocotyl and root elongation in a comparable manner. Thus, we focused on sucrose as triggering substance in ongoing experiments.

### Transport Mutants Differ From a Biosynthesis Mutant

It was shown in 2008 by our group that *int1.1* plants accumulate inositol in their vacuoles due to lacking transport of vacuolar inositol into the cytosol (Schneider et al., 2008). Accordingly, the simplest explanation for reduced hypocotyl and root elongation in *int1.1* mutants would be a reduced presence of cytosolic inositol. Thus, mutants in inositol biosynthesis should display a similar phenotype as the *int1.1* transport mutant. We therefore included the *mips1* mutant (Chen and Xiong, 2010; Luo et al., 2011) in our studies. MIPS1 is the major player in inositol *de novo* biosynthesis; MIPS2 and MIPS3 are isoforms of MIPS1 but play a rather marginal role (Chen and Xiong, 2010; Luo et al., 2011). Analysis of dark-grown WT, *int1.1* and *mips1* seedlings revealed that the transport and the biosynthesis mutant display a comparable phenotype to only some extent (Figure 3A). Hypocotyls of the *int1.1* mutant were – as shown before (Figure 2) – reduced in length on inositol-free medium without and with sucrose, however, sucrose supply enhanced the observed length reduction in a highly significant manner (indicated by white asterisks, Figure 3A). Complementation of the impaired *int1.1* hypocotyl elongation was possible by adding 0.56 mM inositol on medium without sucrose as well as on medium with sucrose. In contrast to that, the hypocotyls of *mips1* seedlings were reduced in length under all conditions, and more importantly, without any enhancing effect of sucrose (Figure 3A). An inositol concentration of 0.56 mM was not sufficient for complementing the *mips1* biosynthesis mutant. These results suggest that the inhibition of hypocotyl elongation (dependent on sucrose) observed in the *int1.1* mutant is not only due to pure inositol deficiency as in the *mips1* mutant. Obviously, there are more factors involved, which rely on sucrose-induction.

The results obtained for light-grown roots were similar, but not identical to those for hypocotyls of dark-grown seedlings. In contrast to hypocotyls, roots of *int1.1* seedlings displayed reduced length compared to WT roots on medium containing 60 mM sucrose and 0 mM inositol only, but not on sugar-free medium (Figure 3B). Nevertheless, comparable to the hypocotyl data, the *int1.1* roots on 60 mM sucrose and 0 mM inositol were significantly shorter than *int1.1* roots on medium without sucrose and inositol, while WT roots benefited from sucrose addition and showed longer roots under sucrose supply (indicated by white asterisks; Figure 3B). The *mips1* mutant seedlings had shorter roots than WT and *int1.1* seedlings under all conditions except on medium supplemented with sucrose and inositol. Comparing the length of *mips1* roots grown on inositol-free medium without and with sucrose revealed also a benefitting effect of sucrose on *mips1* mutants, which is contrary to what was observed for *int1.1* roots (Figure 3B). Thus, we conclude that length reduction in *int1.1* mutants does not or not only depend on inositol deficiency, but also on sucrose-dependent regulatory mechanisms. Importantly, our data suggest that the *de novo* biosynthesis of inositol and its mobilization from the vacuole might support different functional pools of inositol that support different downstream events.

### The Inhibitory Effect of Sucrose Is Dose-Dependent

**FIGURE 4:**
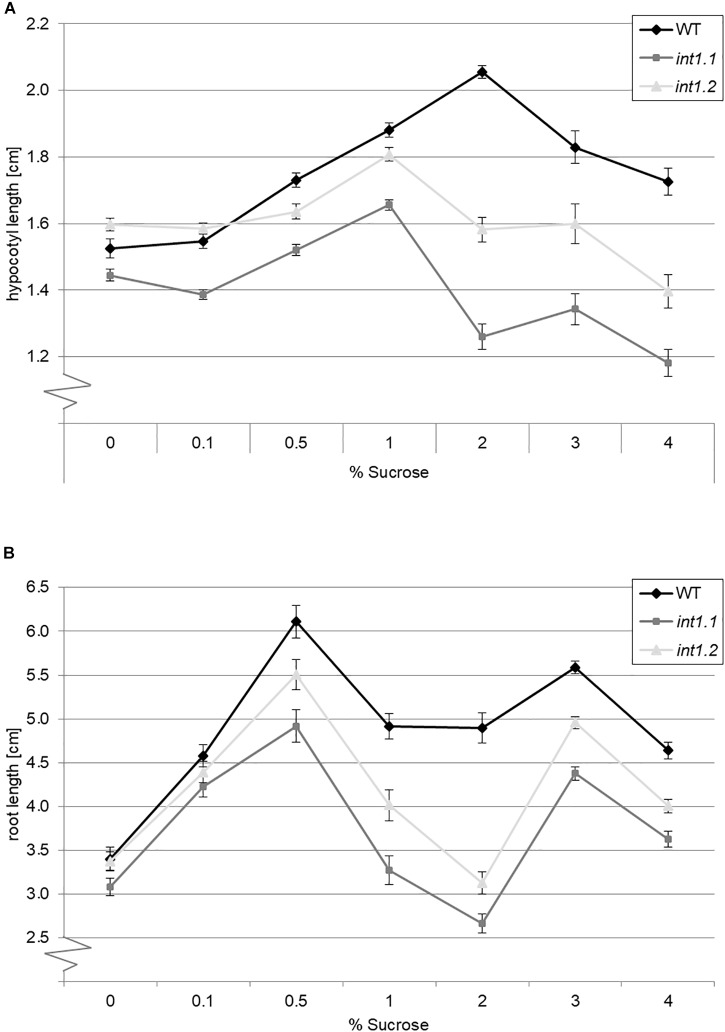
Length of etiolated hypocotyls or light-grown roots on different sucrose concentrations. Seedlings of WT, *int1.1*, and *int1.2* plants were grown in the dark or in the light, respectively, on sucrose concentrations as indicated. **(A)** The hypocotyl length was determined after 7 days; *n* = 43–147 per line and per medium. Error bars = standard error. **(B)** Root lengths of seedlings after 7 days growth under long-day conditions; *n* = 39–54 per line and per medium. Error bars = standard error.

We aimed to find out if the severity of the sucrose-induced length-reduction is depending on the sucrose concentration in the medium, or if the sucrose concentration needs to reach a certain threshold that triggers the inhibitory effect on elongation. Thus we grew WT and *int1.1* mutants as before in darkness and under long-day conditions, but on different sucrose concentrations (0, 0.1, 0.5, 1, 2, 3, and 4%; =3, 15, 30, 60, 90, and 120 mM; Figure 4). We also included the *int1.2* mutant line, which is a T-DNA insertion line with reduced INT1 activity that so far showed an intermediate phenotype between WT and *int1.1* (Schneider et al., 2008). Dark-grown WT and mutant seedlings showed an increase in hypocotyl length with increasing sucrose concentrations from 0 to 1.0% (Figure 4A). While 2% sucrose further promoted elongation in WT hypocotyls, the *int1.1* as well as the *int1.2* mutant showed a severe reduction in hypocotyl length when grown on 2% sucrose. As observed before (Figures 2E, 3A), hypocotyls of *int1.1* seedlings were highly significant shorter on medium containing 2% sucrose compared to those grown on medium without carbohydrate supply (*p* < 0.01). Higher sucrose concentrations (3 and 4%) led to a continually decrease in hypocotyl length in WT seedlings. Nevertheless, WT hypocotyls were still longer when grown on any of the tested sucrose concentrations than on medium without sucrose. In contrast to that, 3% sucrose does not induce a stronger length reduction in *int1* mutant hypocotyls, only 4% of sucrose lead to an even more severe effect than 2% sucrose in both *int1* mutants (resulting in highly significant shorter hypocotyl lengths compared to seedlings grown on sugar free medium; *p* < 0.01; Figure 4A). In summary, *int1* mutant hypocotyls were shorter than WT hypocotyls, with the highest length differences at 2% sucrose and with a more severe length reduction in the *int1.1* knockout mutant than in the *int1.2* mutant, which has residual INT1 activity (Schneider et al., 2008; Figure 4A).

The results obtained for roots of light-grown seedlings were similar (Figure 4B). The *int1* mutant roots were shorter than WT roots on sucrose concentrations from 0.5 to 4%, with a less severe impact of sucrose on the *int1.2* mutant compared to the *int1.1* knockout mutant. The major difference between WT and *int1* mutant root lengths was observed on 2% sucrose, with *int1.1* seedlings having shorter roots than on medium without sucrose supply (*p* < 0.01), confirming previous results (Figure 3B). In line with the results described above, WT roots on 2% sucrose were longer than WT roots on sucrose free medium (Figure 3B). Interestingly, although root growth obviously benefits from sucrose supply, sucrose concentration and root length do not strictly correlate. Concentrations of 0.5 and 3% sucrose induced major root lengths, while the concentrations lying in-between (1 and 2%) had a less positive effect on WT and even an inhibitory effect on *int1* mutant roots. This is contrary to what was observed for hypocotyls, where increasing sucrose concentrations linearly increased hypocotyl lengths up to 2% (WT) or 1% (*int1* mutants), respectively, while higher concentrations lead to a nearly linear decrease of hypocotyl lengths.

As the largest differences in hypocotyl and root length were observed on 2% sucrose, we used this concentration for further experiments.

### Genes of Phosphoinositide Metabolism Are Differently Expressed in Etiolated *int1.1* Hypocotyls

Inositol possesses a multifunctional role in plant metabolism. To understand which metabolic processes rely on altered intracellular inositol distribution combined with sucrose-mediated inhibition of root and hypocotyl elongation we compared the expression levels of several genes encoding for enzymes in inositol metabolism in *int1.1* and WT plants grown under the respective conditions. Because of the plenty of metabolic pathways in which inositol is involved we concentrated on key enzymes in inositol metabolism (indicated in gray boxes in Figure 1). As several isoforms for each key enzyme exist, we focused on target genes with an expression pattern comparable to *INT1*. Expression patterns were figured out using eFP Browser (Winter et al., 2007) and Genevestigator (Hruz et al., 2008).

**FIGURE 5:**
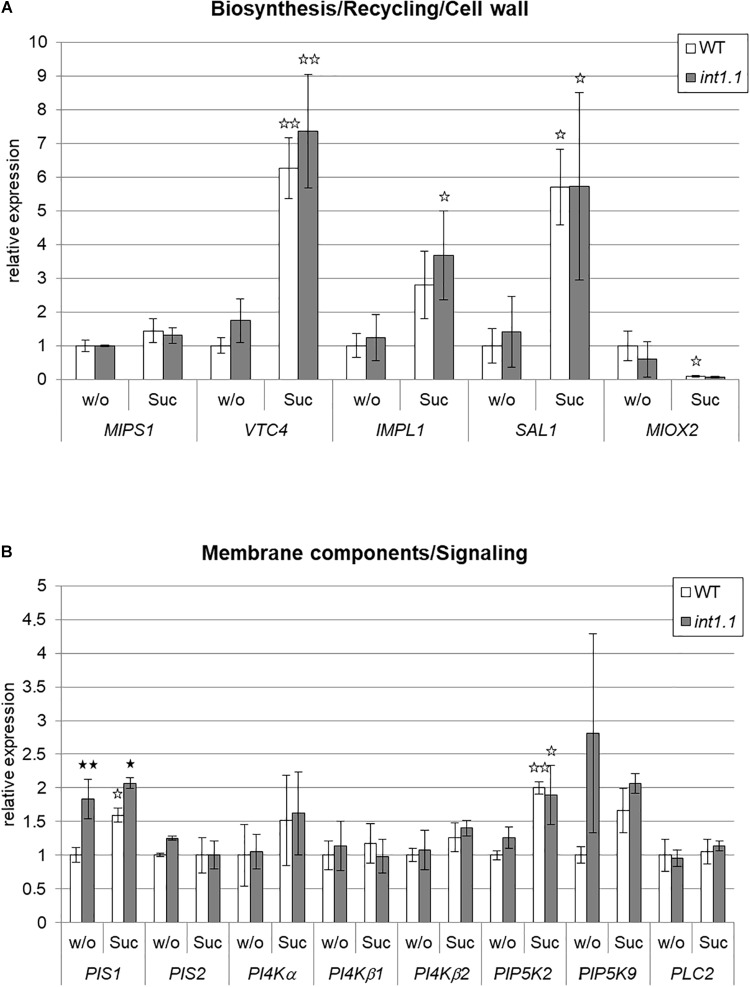
qPCR analysis of genes involved in inositol metabolism in etiolated hypocotyls. Gene expression was analyzed in hypocotyls of 4-day-old etiolated WT and *int1.1* seedlings grown on inositol-free medium without sucrose (=w/o) or with 2% sucrose added (=Suc). **(A)** Genes involved in inositol biosynthesis, inositol salvage pathway and cell wall biosynthesis. **(B)** Genes related to biosynthesis of inositol-containing membrane components and IP3-signaling. Relative gene expression levels were normalized to the reference gene (*UBI10*). Re-adjustment was performed to the expression levels of WT hypocotyls grown on 0 mM sucrose, 0 mM inositol, which were set at 1.0. Mean values of three independent experiments are given; error bars = standard deviation. Differences between genotypes were analyzed using one-way ANOVA followed by Tukey HSD test. Black asterisks indicate a highly significant difference compared to WT on the respective medium, white asterisks indicate a highly significant difference between lines grown on medium without sucrose and the same plant line on medium with sucrose (one asterisk = *p* < 0.05, two asterisks = *p* < 0.01).

In Figure 5 the expression levels of the genes of interest in etiolated *int1.1* hypocotyls compared to WT are given; seedlings were grown on medium with and without sucrose addition. Figure 5A refers to genes involved in inositol biosynthesis, recycling, and cell wall structure; Figure 5B shows expression of candidate genes involved in membrane synthesis and PIP2-derived signaling. For inositol biosynthesis and recycling we observed a general up-regulation of the respective genes (*VTC4*, *IMPL1*, and *SAL1*) by sucrose addition (indicated by white asterisks), but differences between *int1.1* mutant and WT plants did not manifest. Also, for MIOX2, an enzyme involved in cell wall synthesis (Kanter et al., 2005), no differences for gene expression were observed between WT and *int1.1*.

A different result was obtained for enzymes taking part in membrane biosynthesis. The gene for PIS1, one isoform of phosphatidylinositol-synthase, was up-regulated in the *int1.1* mutant under both growth conditions, with a higher factor on medium without sucrose (1.83-fold on 60 mM, 1.30-fold on 0 mM sucrose). Another isoform, *PIS2*, showed no different expression in the mutant on both media. The three analyzed isoforms of phosphatidylinositol 4-kinase (PI4Kα, PI4Kβ1, and PI4Kβ2), the enzyme catalyzing the subsequent reaction in phosphatidylinositide biosynthesis, did also not vary in gene expression. PIP5K9, phosphorylating phosphatidylinositol-4-phosphate (PI4P) to PIP2, shows a strong tendency to have an up-regulated gene expression in the mutant on medium without sugar, although the values did not significantly differ. *PLC2*, encoding the enzyme that generates the second messenger IP3, was not significantly altered.

In roots, variations in gene expression levels between WT and *int1.1* or between different media were only detected for *MIPS1* (Supplementary Figure 2). *MIPS1* in WT shows a higher expression level on medium without sucrose, while sugar supply does not impact the expression level of *MIPS1* in *int1.1* roots (Supplementary Figure 2A).

### Phosphoinositides Are Increased in *int1.1* Hypocotyls

**FIGURE 6:**
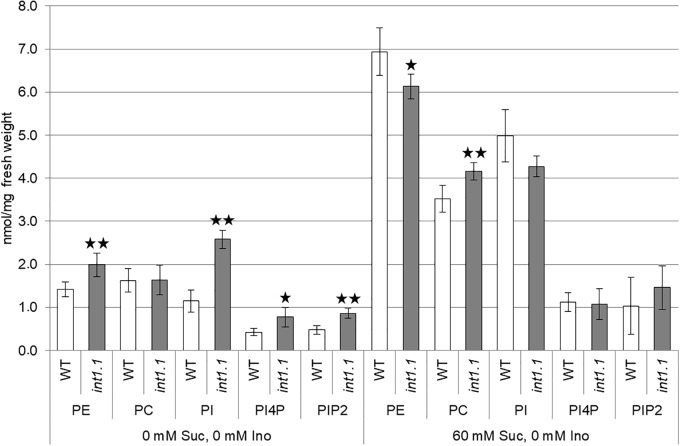
Lipid composition of etiolated hypocotyls. The content of phosphatidylethanolamine (PE), phosphatidylcholine (PC), phosphatidylinositol (PI), phosphatidylinositol 4-phosphate (PI4P), and phosphatidylinositol 4,5-bisphosphate (PIP2) in etiolated hypocotyls of WT and *int1.1* of 7-day-old seedlings grown on 0 mM sucrose, 0 mM inositol, and 60 mM sucrose, 0 mM inositol was determined. Five biological replicates of about 50–80 etiolated seedlings per sample were analyzed. Error bars = standard deviation. Differences between genotypes were analyzed for each membrane component per medium using one-way ANOVA followed by Tukey HSD test. Black asterisks mark a highly significant difference compared to WT (one asterisk = *p* < 0.05, two asterisks = *p* < 0.01).

As some genes encoding enzymes in membrane biosynthesis were up-regulated in etiolated hypocotyls, we analyzed the *int1* transport mutant regarding its membrane composition (Figure 6). The content of phosphatidylinositol (PI), PI4P, PIP2 and of phosphatidylcholine (PC) and phosphatidylethanolamine (PE) of WT and *int1.1* hypocotyls grown on medium with 0 mM sucrose and 60 mM sucrose (each without inositol) were compared. When grown on medium without sucrose, where only minor length differences in elongation occurred, *int1.1* hypocotyls had an about twofold increased content of inositol containing lipids as PI, PI4P, and PIP2. In contrast to that, inositol-derived lipids were not altered under conditions where *int1.1* mutants showed an increased inhibition of hypocotyl elongation (60 mM sucrose, no inositol). These results are contrary to what has been observed in inositol biosynthesis mutants, which possess reduced levels of phosphatidylinositides (Chen and Xiong, 2010; Donahue et al., 2010). Furthermore, it suggests that *int1.1* plants might – at least partly – complement a growth inhibition by increasing inositol containing lipids, but only under non-sucrose conditions. Higher levels of phosphoinositides might rescue a disturbed vesicle trafficking as it was observed for *mips* mutants (Chen and Xiong, 2010; Luo et al., 2011) or might serve as precursor for signaling molecules as, for example, IP3.

### Reduced Hypocotyl Elongation of *int1* Mutants Is Not Linked to IP3 Signaling

**FIGURE 7:**
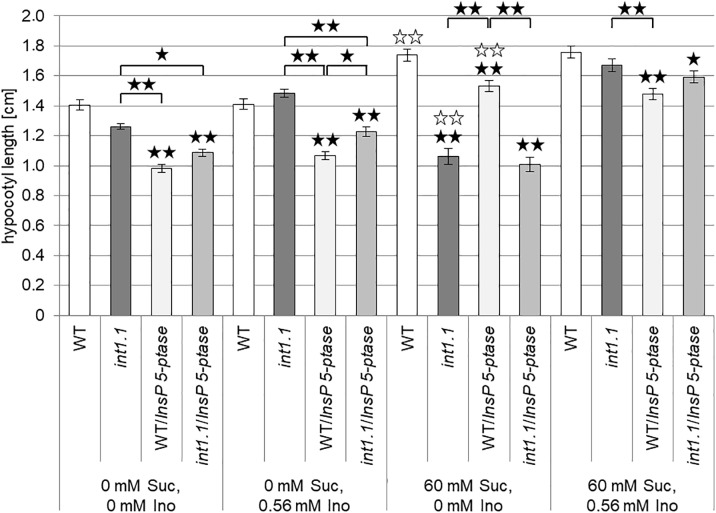
Impact of reduced IP3 content on hypocotyl elongation of dark-grown seedlings. Statistical analysis of 7-day-old etiolated WT and *int1.1* hypocotyls and WT and *int1.1* hypocotyls expressing human *InsP 5-ptase* on media with different sucrose and/or inositol concentrations as indicated. Number of analyzed hypocotyls per line and per medium = 149–180; error bars = standard error. Differences between genotypes were analyzed using one-way ANOVA with *post hoc* Tukey HSD test. Black asterisks indicate a highly significant difference compared to WT (or between two different lines when marked with brackets) grown on the same medium, white asterisks indicate a highly significant difference between lines grown on medium without inositol and without sucrose and the same plant line on medium without inositol, but with sucrose (one asterisk = *p* < 0.05, two asterisks = *p* < 0.01).

IP3 is derived from PIP2, which is one of the inositol containing lipids increased in *int1.1* mutants. A higher PIP2 content was already shown to accompany an increase in IP3 (Perera et al., 1999; DeWald et al., 2001; Im et al., 2014), so we hypothesized that the *int1.1* mutant might have higher levels of IP3 compared to WT when grown without sucrose and that a putative increase or an elevated turnover of IP3 might help to mitigate the elongation inhibition. Hence, we aimed to analyze a potential impact of IP3 on developmental processes in the *int1.1* mutant by constantly reducing the IP3 content. Perera et al. (2006) established an Arabidopsis mutant line expressing the human type I inositol polyphosphate 5-phosphatase gene (*InsP 5-ptase*) under the control of the 35S promoter. These *InsP 5-ptase* plants showed a drastically reduced IP3 content (Perera et al., 2006). In case that a higher level of IP3, potentially derived from the higher PIP2 content, can prevent inhibition of elongation in the *int1.1* mutant on medium without sucrose, this should be no longer possible in *int1.1/InsP 5-ptase* plants. According to this hypothesis, *int1.1/InsP 5-ptase* should show the same strong reduction in hypocotyl length on medium without sucrose as on medium containing sucrose. Thus, we established homozygous *int1.1* and WT lines expressing human *InsP 5-ptase* comparable to those described by Perera et al. (2006). The advantage of using human InsP 5-ptase is its specificity for IP3 without hydrolyzing inositol phospholipids, while Arabidopsis inositol 5-phosphatases are supposed to also accept inositol phospholipids as substrates (Perera et al., 2006). For analysis we used plant lines with a similar *InsP 5-ptase* expression level. We compared hypocotyl elongation of WT and *int1.1* seedlings with WT and *int1.1* seedlings expressing *InsP 5-ptase* on 0 mM sucrose, 0 mM inositol and 60 mM sucrose, 0 mM inositol (Figure 7). Etiolated WT and *int1.1* seedlings not expressing *InsP 5-ptase* showed the same results as described above: a significant length reduction of *int1.1* hypocotyls on medium with 60 mM sucrose compared to WT (and compared to *int1.1* hypocotyls on medium without sucrose, indicated by white asterisks) (Figure 7). WT and *int1.1* seedlings expressing *InsP 5-ptase* had principally shorter hypocotyls compared to their parent lines without *InsP 5-ptase* expression (Figure 7). Nevertheless, on plates containing 60 mM sucrose the same effect could be observed as for the origin plants lines: sucrose induced a strong length reduction in *int1.1* hypocotyls, which could be complemented by addition of 0.56 mM inositol. Hypocotyls grown on medium without sucrose did not confirm our hypothesis stated at the beginning of this paragraph. Thus, we could not link the sucrose-induced length reduction in *int1.1* seedlings to IP3.

### Cell Elongation Is Inhibited in *int1.1* Mutants

**FIGURE 8:**
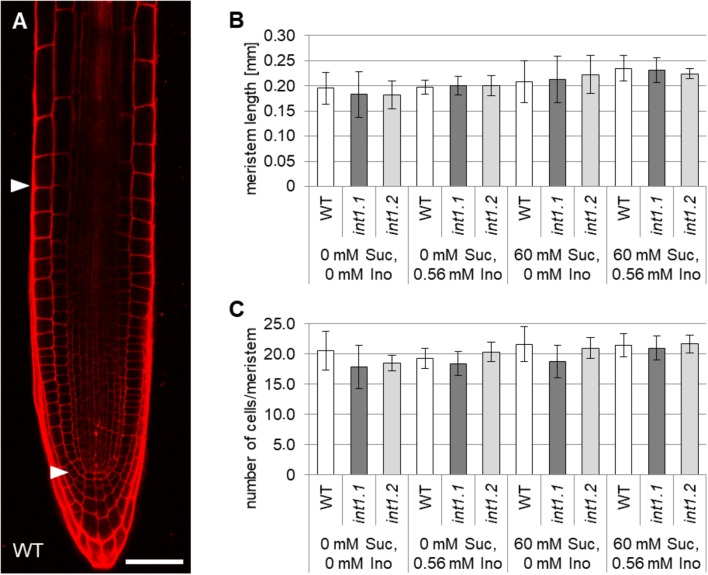
Analysis of root apical meristems in light-grown WT, *int1.1*, and *int1.2* seedlings. **(A)** WT root stained with propidium iodide. Arrowheads mark the proximal and distal border of the meristem. Scale bar = 50 μm. **(B)** Statistical analysis of meristem lengths in 7-day-old WT, *int1.1* and *int1.2* roots grown on media with inositol and sucrose concentrations as indicated. Number of roots analyzed per plant line = 6–12; error bars = standard deviation. **(C)** Quantification of cells per meristem per plant line on medium as indicated. Number of analyzed roots per line = 6–12; error bars = standard deviation. Potential differences between genotypes were analyzed using one-way ANOVA with *post hoc* Tukey HSD test.

**FIGURE 9:**
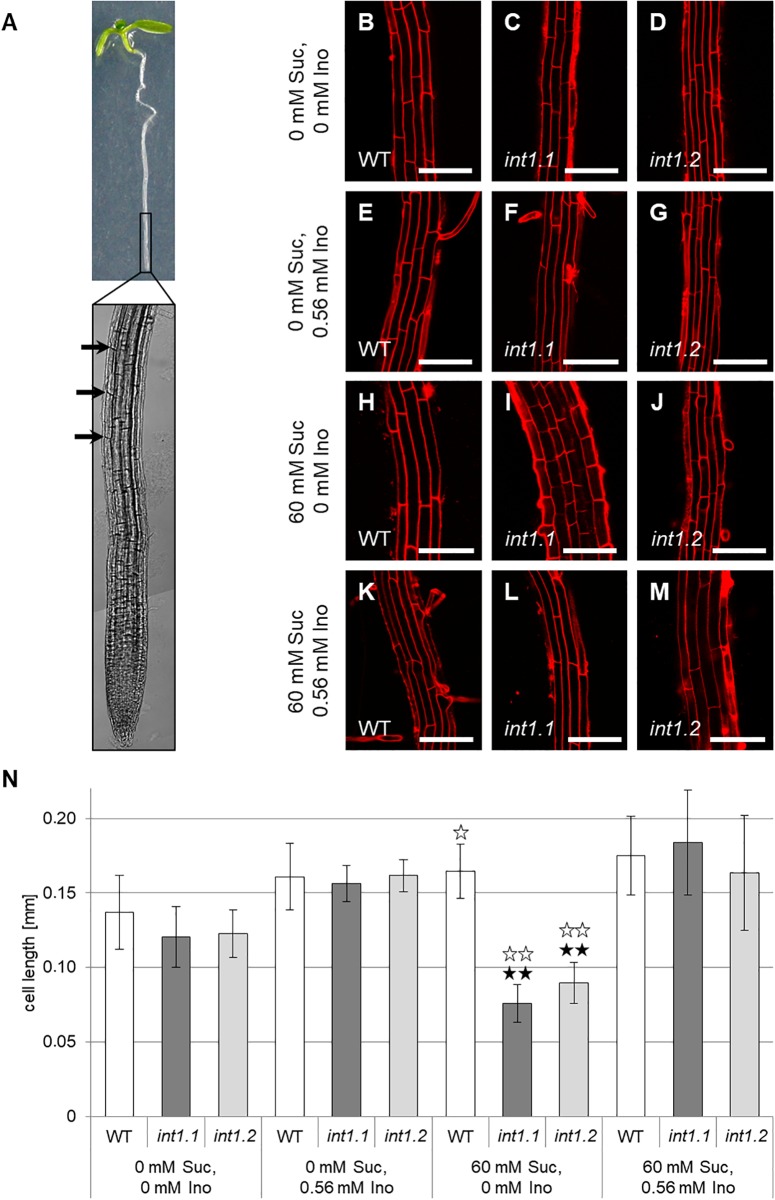
Cell lengths of fully elongated root epidermis cells of WT, *int1.1*, and *int1.2* plants grown on different media. **(A)** Exemplary seedling with root tip enlarged. Black arrows indicate the first fully elongated cells above root tip from where on length analysis was performed. Details of roots of 7-day-old seedlings on medium with 0 mM sucrose, 0 mM inositol **(B–D)**, 0 mM sucrose, 0.56 mM inositol **(E–G)**, 60 mM sucrose, 0 mM inositol **(H–J)** and on medium with 60 mM sucrose, 0.56 mM inositol **(K–M)** of WT **(B,E,H,K)**, *int1.1* **(C,F,I,L)**, and *int1.2* **(D,G,J,M)** plants. Scale bars represent 100 μm. **(N)** Statistical representation of epidermis cell lengths on different sucrose and inositol concentrations. Differences between genotypes were analyzed using one-way ANOVA with *post hoc* Tukey HSD test. Black asterisks indicate a highly significant difference compared to WT on the respective medium (one asterisk = *p* < 0.05, two asterisks = *p* < 0.01); white asterisks indicate a highly significant difference between seedlings on medium with 0 mM sucrose, 0 mM inositol and the respective plant line on medium with 60 mM sucrose, 0 mM inositol (two asterisks = *p* < 0.01). Number of cells analyzed per root = 8–37; roots per line and per medium: *n* = 6–12, error bars = standard deviation.

Possible reasons for a reduction in root lengths could be defective activity of the root apical meristem, or a disturbance of cell elongation (Thomann et al., 2009). Hence, we analyzed the length and cell number of the root apical meristem and compared the lengths of cells in the root elongation zone. Root apical meristem lengths, cell numbers per meristem and lengths of epidermal cells in elongation zones were analyzed in 7-day-old roots of WT, *int1.1* and *int1.2* seedlings on medium with 0 or 60 mM sucrose and with and without inositol (Figures 8, 9). No differences were observed for meristem lengths as well as for cell numbers per meristem between WT and *int1* mutant seedlings, regardless of the sucrose and/or inositol supply (Figures 8B,C).

In contrast to that, cell elongation was strongly impaired in *int1* mutant roots, but only in presence of 60 mM sucrose (Figures 9H–J,N). Cell lengths did not vary on medium without sucrose or if inositol was supplied (Figures 9B–G,K–N). Comparing epidermis cells in the elongation zones of roots grown on 60 mM sucrose, 0 mM inositol to those on 0 mM sucrose, 0 mM inositol revealed that WT cells were more elongated under sucrose supply, while *int1.1* and *int1.2* cells were highly significant shorter when sucrose was added (*p* < 0.01). This falls into line with the results obtained for overall root length (Figure 3B).

**FIGURE 10:**
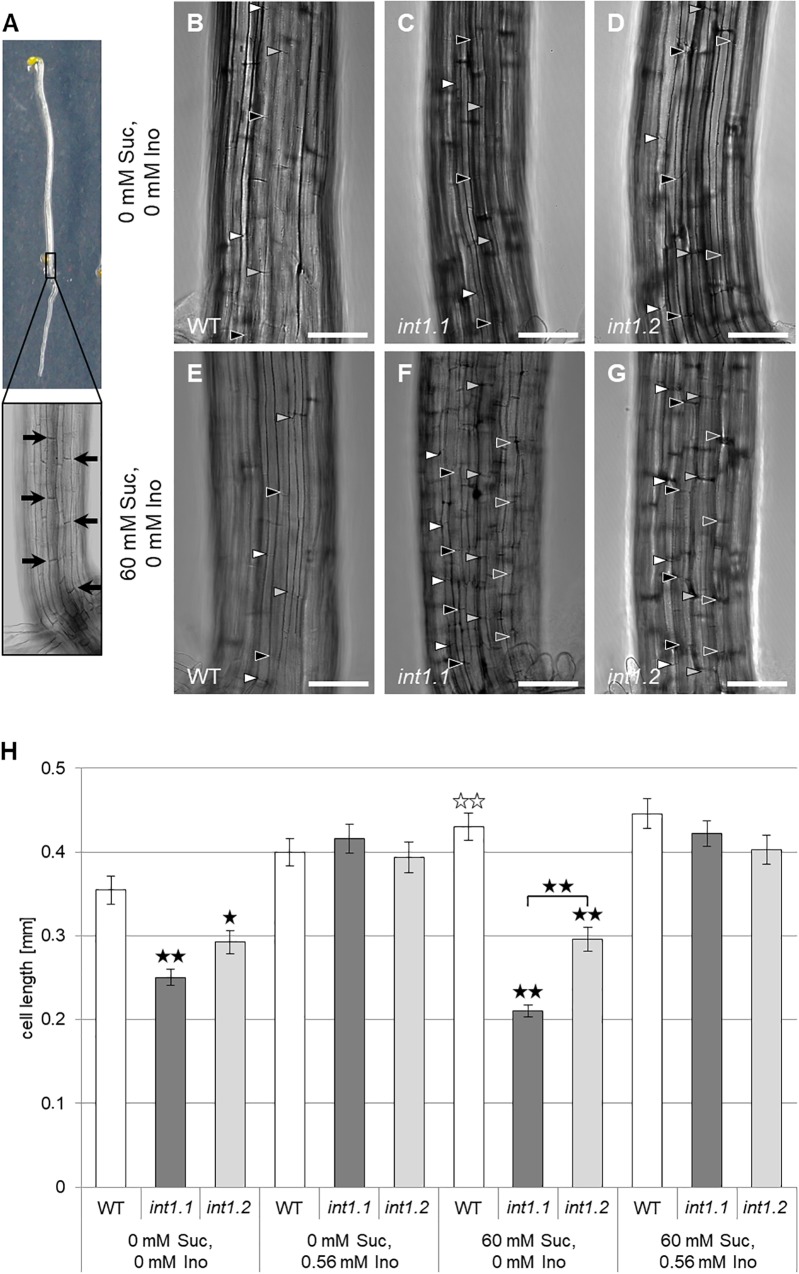
Cell length in etiolated hypocotyls of WT, *int1.1*, and *int1.2* plants on different media. **(A)** Exemplary etiolated seedling with basal hypocotyl region enlarged. Black arrows indicate the fully elongated cells above root-to-shoot transition used for length analysis. Details of etiolated hypocotyls of 4-day-old seedlings on medium with 0 mM sucrose, 0 mM inositol **(B–D)** and on medium with 60 mM sucrose, 0 mM inositol **(E–G)** of WT **(B,E)**, *int1.1* **(C,F)**, and *int1.2* **(D,G)** plants. Arrowheads indicate the apical and basal cell walls of elongating hypocotyl cells, with same colors marking adjacent cells of a vertical row. Bars represent 100 μm. **(H)** Statistical representation of cell lengths on different sucrose and inositol concentrations. Differences between genotypes were analyzed using one-way ANOVA with *post hoc* Tukey HSD test. Black asterisks indicate a highly significant difference compared to WT (or between two different lines when marked with brackets) on the respective medium (one asterisk = *p* < 0.05, two asterisks = *p* < 0.01); white asterisks indicate a highly significant difference between hypocotyls grown on medium with 0 mM sucrose, 0 mM inositol and hypocotyls of the respective plant line on medium with 60 mM sucrose, 0 mM inositol (two asterisks = *p* < 0.01). Number of cells per line and per medium: *n* = 42–64, error bars = standard error.

We also analyzed the cell length of etiolated hypocotyls of *int1.1* and WT plants on medium with and without sucrose and with and without inositol supplement (Figure 10). On growth media without inositol supply hypocotyls of both mutant lines displayed significantly less elongated cells than WT hypocotyls (Figures 10B–H). As expected after previous results, the *int1.2* line showed an intermediate cell length between the WT and *int1.1* line (Figures 10G,H). The reduction of cell elongation could be complemented by adding inositol (0.56 mM; Figure 10H). Overall, the data indicate that meristem activity is normal, whereas the elongation of root and hypocotyl cells is impaired in the *int1.1* and *int1.2* mutants.

### Vacuolar Turgor Is Not Altered in *int1.1* Mutants

As cell elongation is a vacuole-driven process, the data suggest that the lack of vacuolar inositol exporters might impair the build-up of turgor pressure for elongation. Thus, we analyzed the vacuolar morphology of *int1.1* and WT root cells using membrane dye MDY-64 under normal and under hypertonic conditions by adding different concentrations of mannitol (Supplementary Figure 3). No morphological differences could be observed between WT and *int1.1* under normal conditions (Supplementary Figures 3A–D). Addition of increasing concentrations of mannitol resulted in progressing plasmolysis, but without apparent differences in WT and *int1.1* cells (Supplementary Figures 3E–L). This indicates that vacuoles of WT and *int1.1* possess a comparable turgor pressure.

### Cell Wall Composition of Etiolated *int1* Hypocotyls Differs From WT

**FIGURE 11:**
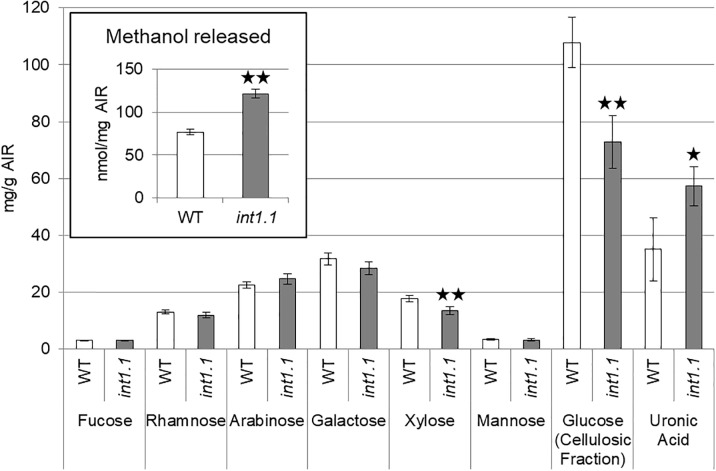
Cell wall analysis of 7-day-old etiolated hypocotyls grown on MS medium with 60 mM sucrose, 0 mM inositol. Carbohydrate composition was analyzed by HPAEC-PAD. Methanol released (inset in diagram) was measured after saponification of the AIR. Statistic relevant differences were analyzed using one-way ANOVA with *post hoc* Tukey HSD test. Black asterisks indicate a highly significant difference compared to WT (one asterisk = *p* < 0.05, two asterisks = *p* < 0.01), error bars = standard deviation.

Pectins, hemicelluloses, and cellulose form the cell wall and alterations of their amount, composition or post-synthesis modification can severely impact cell elongation. Carbohydrate composition and the state of methyl-esterification of etiolated seedlings grown on MS medium containing 60 mM sucrose, 0 mM inositol were analyzed (Figure 11). Levels of xylose and cellulose-derived glucose were lower, while uronic acid of the ammonium oxalate extracted fraction was higher in etiolated *int1.1* seedlings. This indicates a lower content of cellulose, altered xyloglucan structure and higher amount of homogalacturonan in the mutant compared to the WT. Finally, the saponification of the cell wall did not reveal a significant change of the methyl-esterification degree of homogalacturonan between the two genotypes (diagram inset, Figure 11; ratios of methanol/uronic acids: WT = 2.39 ± 0.85, *int1.1* = 2.14 ± 0.24). No differences were observed for the carbohydrate composition of seedling roots grown under long-day conditions (Supplementary Figure 4).

### Etiolated *int1* Hypocotyls Have Elevated Levels of Inositol and Sugars

**FIGURE 12:**
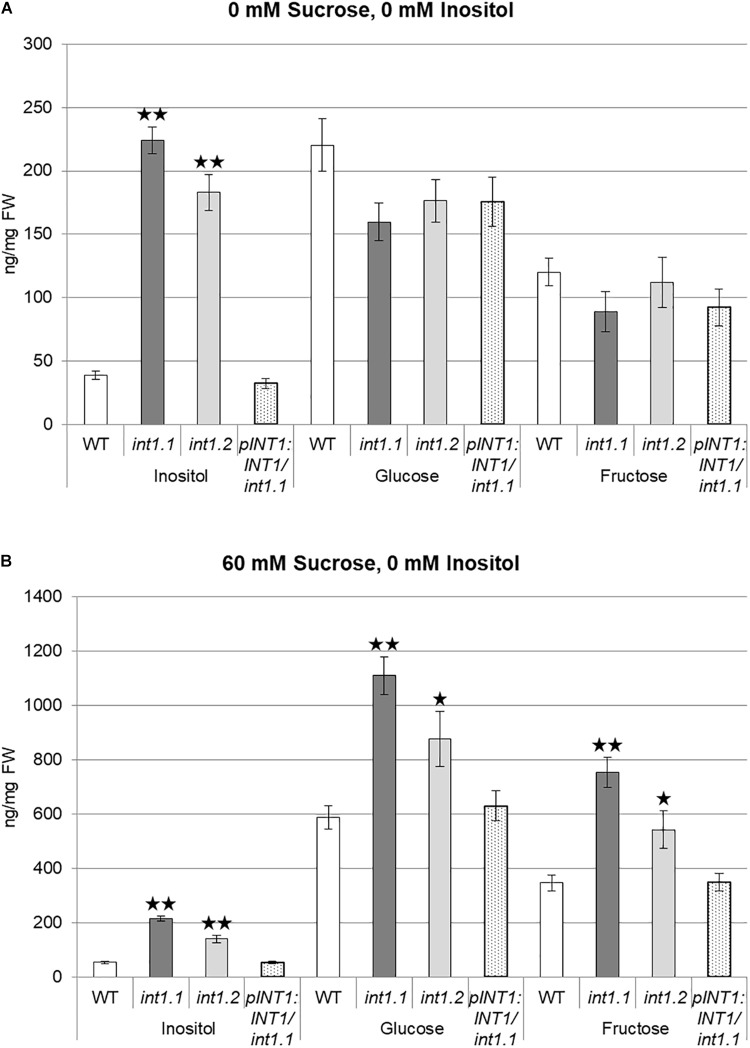
Inositol, glucose, and fructose content of dark-grown seedlings. WT, *int1.1*, *int1.2*, and *pINT1:INT1*/*int1.1* seedlings were grown for 7 days in the dark on MS medium containing 0 mM sucrose, 0 mM inositol **(A)** or 60 mM sucrose, 0 mM inositol **(B)**. Sugar and sugar alcohol content was analyzed by ion chromatography. Four to eight biological replicates of 22–58 seedlings per sample were analyzed. Differences between genotypes were analyzed using one-way ANOVA with *post hoc* Tukey HSD test. One asterisk indicates a significant difference of *p* < 0.05, two asterisks indicate for *p* < 0.01. Error bars = standard error.

During the initial characterization of *int1* mutants it was shown that rosette leaves of *int1* plants had elevated levels of inositol but did not vary from WT for glucose or fructose content (Schneider et al., 2008). This goes in line with INT1 activity – proton-driven inositol transport into the cytosol – and therefore accumulation of inositol within the vacuole in *int1* mutants. Here, we analyzed etiolated seedlings for inositol and sugar content. When grown on MS medium without any carbohydrate supply (0 mM sucrose, 0 mM inositol, Figure 12A), inositol levels were strongly increased in *int1* plants compared to WT, but glucose and fructose contents did not vary in a significant way. Surprisingly, etiolated *int1* seedlings grown on medium with 60 mM sucrose and 0 mM inositol contained not only significantly higher amounts of inositol, but also of glucose and fructose (Figure 12B). Inositol, glucose, and fructose levels of a complementation line (*int1.1* plants expressing the *INT1* coding sequence under control of the *INT1* promoter, named *pINT1:INT1/int1.1*) did not differ from those in WT on both growth media, confirming that not only elevated inositol levels, but also the increase of glucose and fructose are attributed to the absence of INT1.

Additionally to etiolated seedlings grown in the dark also roots of seedlings grown under long-day conditions were examined for inositol and sugar content. In contrast to etiolated seedlings, *int1* roots showed approximately doubled inositol levels, but no significant differences for glucose or fructose, independent of the growth medium (Supplementary Figure 5).

### PIN2 Localization Is Not Altered in *int1.1* Roots

For *mips1* mutants alterations in PIN1 and PIN2 protein localization were discussed to be causative for shorter roots in *mips1* seedlings (Chen and Xiong, 2010; Luo et al., 2011). As the coalition of vacuoles during turgor build-up is furthermore dependent on auxin (Löfke et al., 2015), we hypothesized that auxin might contribute to the phenotype of the *int1* mutants and crossed the *pPIN2:PIN2-GFP* allele (Xu and Scheres, 2005) into the *int1.1* background. Roots of PIN2-GFP and homozygous *int1.1*xPIN2-GFP seedlings were analyzed after growth on medium without inositol and with or without sucrose. The fungal toxin BFA inhibits the re-cycling from endosomes to the plasma membrane. Subsequently, BFA addition results in an accumulation of PIN2-GFP in so-called BFA compartments (Geldner et al., 2001; Kleine-Vehn et al., 2008). Impaired endocytosis would result in delayed appearance of PIN2-GFP labeled BFA compartments in *int1.1* mutant background. PIN2-GFP cycling was monitored directly after BFA addition and after 30, 60, and 90 min incubation time (Supplementary Figure 6).

PIN2-GFP visualized endocytosis in *int1.1* roots did not differ from that observed in WT roots at any examined time point. After washout for 1 or 2 h polar PIN2-GFP localization was equally re-established in WT and *int1.1* background (Supplementary Figure 6).

### Vesicle Trafficking Is Not Impaired in *int1.1* Seedlings

Phosphoinositides as PI4P and PIP2 can regulate membrane trafficking and vesicle formation (Jost et al., 1998; Di Paolo and De Camilli, 2006; McNiven and Thompson, 2006). Hence, another explanation for impaired cell elongation might be that vesicle trafficking is restricted under sucrose supply – without being reflected in phosphoinositide content. We assumed that mutants on carbohydrate-free medium could complement this effect by increased membrane turnover, indicated by an increased content of phosphoinositides. Thus, we analyzed vesicle trafficking using the membrane dye FM4-64. Endocytosis can be monitored almost immediately after FM4-64 addition, but short-time staining of hypocotyl cells was not sufficient to allow the membrane stain to permeate the strongly cutinized cell walls of etiolated hypocotyls. Therefore, we had to analyze FM4-64-visualized vesicle trafficking in roots (Supplementary Figure 7).

In line with unaltered PIN2-cycling we could not observe any differences in endocytosis of FM4-64 stained vesicles. After staining with FM4-64 for 5 min on ice, first FM4-64 marked vesicles could be observed sporadically already after 15 min, independent from plant line and growth medium (Supplementary Figure 7). After 30 and 60 min a comparable turnover of FM4-64 stained vesicles was detectable without any apparent differences between WT and *int1.1* roots.

## Discussion

We showed that sucrose induces a strong inhibition in cell elongation in roots as well as in hypocotyls of *int1* mutants. In *int1* mutants transport of vacuolar *myo*-inositol into the cytoplasm is disturbed. Although *myo*-inositol is the physiological substrate of all plant inositol transporters characterized so far, additional substrates as, for example, D-*chiro*-inositol, *scyllo*-inositol and methylated inositols as ononitol and pinitol are accepted (for overview see Schneider, 2015). Although we cannot exclude transport activity of INT1 for other substrates than *myo*-inositol, we can clearly state, based on our data, that the observed inhibition in root and hypocotyl elongation depends on *myo*-inositol and not on other putative substrates. First, the reduction in hypocotyl as well as root length in *int1* seedlings could be complemented by addition of inositol (Figures 2, 3, 9, 10). Second, it was already shown in earlier work, that, in fact, *myo*-inositol accumulates in *int1.1* mutants (Schneider et al., 2008). Moreover, the data presented in this work reveal increased inositol levels in etiolated *int1* seedlings and in *int1* roots under all conditions analyzed (the increase of glucose and fructose occurring only under certain conditions will be discussed below). Hence, missing re-import of vacuolar *myo*-inositol into the cytoplasm is responsible for reduced cell elongation in *int1* roots and hypocotyls. Consequently, the obvious conclusion at first view is that reduced cell elongation is due to *myo*-inositol depletion in *int1* mutants, as it was stated before (Schneider et al., 2008). However, several new pieces of information now argue against this explanation. Most important: in *int1* mutants a strong inhibition of cell elongation only occurs under sucrose supply. This is in contrast to WT seedlings, where etiolated hypocotyls as well as roots in principle benefit from sucrose addition (Freixes et al., 2002; Takahashi et al., 2003; Zhang et al., 2010; Kircher and Schopfer, 2012). In accord with this, sucrose supply in the growth medium showed a positive impact on WT seedlings, while roots and hypocotyls of *int1.1* seedlings were significantly shorter on medium with sucrose than on sugar-free medium (Figures 3, 4). Additionally, it has to be kept in mind that the *int1* transport mutants are still capable of an intact *de novo myo*-inositol biosynthesis. Low inositol levels were shown to induce *MIPS* expression in Arabidopsis (Johnson and Sussex, 1995). Hence a reduced cytoplasmic inositol concentration should result in an elevated *MIPS* expression level. However, expression of *MIPS1*, the most important of three MIPS isoforms in Arabidopsis (Donahue et al., 2010), did not vary between WT and mutant neither in etiolated hypocotyls nor in roots on both growth media (Figure 5 and Supplementary Figure 2). Particularly on medium with carbohydrate supply we would expect that lack of recycled vacuolar inositol could be compensated or at least buffered by cytoplasmic inositol biosynthesis.

Of course, INT1-transported *myo*-inositol might contribute to an overall *myo*-inositol pool that serves as source for inositol-related metabolic pathways and biosynthesis might not be sufficient to keep the needed inositol levels if INT1 is missing. Considering this, we would expect that a length reduction in *int1* mutants would predominantly occur on medium without carbohydrate supply, where precursors for inositol biosynthesis are limited. Apart from this the *int1* mutant would resemble the *mips1* mutant, very likely in an attenuated mode. But *int1* and *mips1* seedlings behave differently in several ways: in contrast to *int1* mutants, length reduction in etiolated hypocotyls and roots of light-grown *mips1* mutants was not enhanced by sucrose (Figure 3). Moreover, *int1* hypocotyls showed under growth conditions without sucrose an increased content of phosphoinositides or no differences on sucrose-supplied medium, respectively (Figure 6), while a reduction in phosphoinositide levels was described for *mips1* seedlings (Chen and Xiong, 2010; Donahue et al., 2010). Also, an impaired membrane trafficking as well as altered localizations of PIN1 and PIN2 were described for *mips1* plants (Chen and Xiong, 2010; Luo et al., 2011), but not observed in *int1.1* seedlings (Supplementary Figures 6, 7). Additional defects of *mips1* mutants as wrinkled seeds (Donahue et al., 2010) and a distorted root cap organization (Chen and Xiong, 2010) did not appear in *int1* lines either (Schneider et al., 2008). As the phenotypic appearance of *mips1* mutants is clearly due to disturbed inositol biosynthesis, we set the *mips1* phenotype as a norm for inositol depletion. With the *int1* mutant showing drastic phenotypic differences compared to *mips1* plants, we can exclude that the observed inhibition in cell elongation is solely due to limited inositol availability. It rather indicates that *myo*-inositol transported by INT1 strikes a different metabolic or signaling pathway than *myo*-inositol derived via biosynthesis.

Yeats et al. (2016) recently described a sugar-induced enhancement of a short hypocotyl phenotype in the *shaven3 shaven3-like1* (*shv3svl1*) double mutant similar to the *int1* phenotype. SHV3 is a glycosylphosphatidyl-anchored protein with weak similarity to glycerophosphodiester phosphodiesterase and PI-specific phospholipase C enzymes, but so far, the respective catalytic activity could not be proven (Hayashi et al., 2008; Yeats et al., 2016). A double knockout of *SHV3* and its homolog *SVL1* resulted in reduction of cellulose content and hypocotyl length (Hayashi et al., 2008). Yeats et al. (2016) observed that sucrose addition of 60 mM (2%) strongly enhanced the cellulose deficiency as well as the reduction of hypocotyl length. Uptake of sucrose via sucrose transporter SUC1 was strongly increased in *shv3svl1* hypocotyls, which was visualized using the fluorescent sucrose analog esculin. This was accompanied by accumulation of starch granules in *shv3svl1* hypocotyls. Yeats et al. (2016) concluded that increased intracellular sucrose concentrations subsequently affected carbon partitioning between cellulose and starch, shifting the metabolism from cellulose to starch synthesis, via an indirect impact of SHV3 and SVL1. According to these data, we analyzed if missing inositol transport via INT1 is similarly involved in carbon partitioning as described for *shv3svl1* mutants. Thus, we performed Lugol staining (Supplementary Figure 8) as well as esculin uptake assays (Supplementary Figure 9) with WT and *int1* seedlings under the respective growth conditions. Neither an enhanced accumulation of starch granules (Supplementary Figure 8) nor an increased esculin uptake (Supplementary Figure 9) was detectable in etiolated *int1* seedlings. In conclusion, inhibition of cell elongation in *int1* seedlings is in all probability not due to a shifted carbon usage in favor for starch instead of cellulose as observed for *shv3svl1* mutants.

Nevertheless, we observed alterations in the cellulose content at least in etiolated hypocotyls (Figure 11). Additionally, xylose was also decreased in *int1.1* mutants, suggesting an altered structure of xyloglucan. Moreover, the cell wall of *int1.1* mutant appeared enriched in homogalacturonan compared to the WT. Such an increase in pectins has been observed to accompany cellulose-deficiency before (Peng et al., 2000; His et al., 2001; Robert et al., 2004). Although *myo*-inositol can contribute to the synthesis of the pectin precursor molecule UDP-glucuronic acid via the MIOX pathway (Kanter et al., 2005), the pleiotropic effects also on cellulose and xyloglucan content suggest a regulatory role of INT1-translocated inositol instead of a direct metabolic impact. As turgor pressure is comparable in WT and *int1.1* cells (Supplementary Figure 3), and having in mind that cellulose deficient mutants and plants treated with an inhibitor of cellulose biosynthesis displayed strong altered hypocotyl elongation (Mouille et al., 2003), alterations in cell wall composition, particularly the loss of cellulose, appears to be the limiting factor for cell elongation in *int1* mutants grown on sucrose-containing medium. Interestingly, *int1* plants do not show an obvious radial swelling as commonly described for cellulose-deficient mutants (Fagard et al., 2000; Williamson et al., 2001; Robert et al., 2004; Roudier et al., 2005).

The misdistribution of vacuolar inositol is difficult to connect with altered cell wall composition on first view. Anyhow, a regulatory connection might exist between cell wall and vacuolar lumen. The mutation of a wall-associated receptor-like kinase (WAK) resulted in reduced activity of a vacuolar invertase und subsequently in impaired cell elongation in Arabidopsis (Kohorn et al., 2006). A knockout of a vacuolar invertase, in turn, resulted in reduced glucose and fructose content in the respective *inv-7* mutant, accompanied by less elongated root cells (Wang et al., 2010). This is in contrast to what we observed in *int1* hypocotyls grown on 60 mM sucrose, which have increased glucose and fructose levels (Figure 12), but a strongly inhibited cell elongation (Figure 10). The increase of glucose and fructose levels was observed only in etiolated hypocotyls and only under sucrose supply, while inositol levels were constantly elevated in all *int1* samples (Figure 12 and Supplementary Figure 5; Schneider et al., 2008). Moreover, glucose and fructose were excluded as potentially transported substrates for INT1 before (Schneider et al., 2008). Thus, the increase of glucose and fructose in etiolated seedlings grown on 60 mM sucrose are unlikely to directly depend on missing INT1 activity, but rather a secondary effect. It is difficult to state if the increase in glucose and fructose content is a result of impaired elongation of the respective cells, i.e., metabolites for cellulose biosynthesis were provided, but cannot be used and thus accumulate, or if the elevated levels of glucose and fructose are causative for the altered cellulose content and subsequently for the inhibition of cell elongation in etiolated hypocotyls. Nevertheless, as we did neither observe an increase in glucose or fructose (Supplementary Figure 5) nor alterations in cell wall composition (Supplementary Figure 4) in *int1.1* roots, misdistribution of vacuolar inositol might impair cell elongation in roots by a different process than in etiolated hypocotyls.

We analyzed the cycling of PIN2-GFP and endocytotic membrane turnover using FM4-64 but did not observe any differences between WT and *int1* mutants. So far, we cannot connect the altered intracellular inositol distribution in the *int1.1* mutant to auxin signaling as it was described for mutants impaired in inositol metabolism (Wang et al., 2009; Chen and Xiong, 2010; Zhang et al., 2011; Mei et al., 2012; Ischebeck et al., 2013; Tejos et al., 2014). Nevertheless, we cannot exclude an impact on phytohormone homeostasis via a regulatory mechanism. For example, the auxin receptor TIR1 contains inositol hexa*kis*phosphate as structural co-factor (Tan et al., 2007). In addition, the F-box protein COI, mediating jasmonate signaling, interacts with inositol penta*kis*phosphate (Sheard et al., 2010). Hence, also inositol itself might conceivably act as co-factor or signal-inducing molecule. However, according to the fact that *int1* mutants display a different phenotype than *mips1* mutants, discrimination between INT1-transported inositol and newly synthesized inositol within the plant cell would be required. This discrimination could be theoretically possible as INT1-mediated transport of inositol probably leads to a locally higher concentration of inositol directly at the vacuolar membrane, providing inositol as interacting molecule for a hypothetical (very likely tonoplast-localized) target protein. This target protein might act as regulator in a (yet not identified) signaling pathway leading to inhibition of cell elongation under sucrose supply.

The connection between sucrose supply, impaired inositol transport and inhibition of cell elongation is not easy to link at a glance. Growing seedlings on different sucrose concentrations revealed that a concentration of 60 mM (2%) induced major length differences between WT and *int1* mutants in etiolated hypocotyls as well as in roots of light-grown plants, with less severe length reductions on lower and higher sucrose concentration (Figure 4). This implicates that *int1* mutants are not generally more sensitive to sucrose, but that sucrose at a certain concentration – combined with impaired intracellular inositol transport – triggers a strong inhibition of cell elongation in roots as well as in hypocotyls, in the latter very likely caused by reduced cellulose content.

During our work we speculated that the observed increase in phosphoinositides in *int1.1* hypocotyls grown on medium without sugar might help to rescue the inhibition in cell elongation that occurs under sucrose supply. We hypothesized two possible mechanisms: increasing inositol-containing membrane components (i) might rescue an impaired vesicle trafficking or (ii) might result in higher IP3 levels. An increase in IP3 might in turn complement the inhibition of cell elongation via a signaling course. However, we could not detect differences in vesicle trafficking, making hypothesis (i) obsolete. To analyze an impact of putatively increased IP3 content we introduced the human *InsP 5-ptase*, which was shown before to severely reduce the IP3 content (Perera et al., 2006). According to our hypothesis we expected that dark-grown *int1.1* hypocotyl on sucrose-free medium would then display the same reduced hypocotyl length as those grown on 2% sucrose. As this was not the case we would rather exclude a connection between our observed inhibition in cell elongation and IP3 signaling.

Increase of phosphoinositides on sugar-free medium on the one hand and enhanced inhibition of cell elongation might also underlie completely different regulatory mechanisms with transported inositol as key molecule. It was shown several times before that addition of carbohydrates impacts and alters gene expression in Arabidopsis (Aghdasi et al., 2008; Lee et al., 2012; Lilley et al., 2012; Rottmann et al., 2016), which might result in providing different acceptor proteins for INT1-transported inositol. Our qPCR analysis revealed that several genes related to inositol metabolism were differentially expressed under sucrose supply (Figure 5). Interestingly, the expression of *PIP5K*, encoding a phosphatidylinositol monophosphate 5-kinase, does not vary between WT and *int1* (Figure 5 and Supplementary Figure 2). PIP5K9 interacts with a cytosolic invertase and overexpression of *PIP5K9* resulted in shorter roots and less elongated cells (Lou et al., 2007). Expression of *PIP5K9* was not up-regulated in *int1* mutants under sucrose supply, so the inhibition of cell elongation observed in *int1* does not involve a PIP5K9-dependent regulation.

In contrast to *PIP5K9*, expression of *VTC4*, *IMPL1*, and *SAL1* (all involved in recycling inositol from inositol phosphates; Ishitani et al., 1996; Quintero et al., 1996; Lou et al., 2007; Torabinejad et al., 2009; Robles et al., 2010; Dieck et al., 2012; Nourbakhsh et al., 2015) is highly up-regulated under sucrose supply in etiolated hypocotyls, indicating that more inositol from recycling processes in the cytoplasm is available additionally to biosynthesized inositol under sucrose supply. One would expect that this putative increase in re-filling the cytosolic inositol pool could complement lacking delivery of vacuolar inositol via INT1. In contrast, *int1* mutants are strongly impaired in cell elongation under sucrose supply, a growth conditions where *de novo* biosynthesis and recycling processes should be able to provide sufficient *myo*-inositol to compensate missing access to vacuolar *myo*-inositol. This further supports the consideration that the plant cell can discriminate between inositol molecules derived from different sources and that INT1-transported inositol rather has a signaling than metabolic function. But putative acceptor protein(s) for INT1-delivered inositol remain to be elucidated at the moment.

Although the mechanism of regulation is unclear, we can state that INT1-transported inositol impacts cell elongation in a sucrose-dependent manner. Various sugar-induced regulation processes involved in plant cell expansion are known up to now (reviewed for example in Wang and Ruan, 2013). As we can rule out a simple depletion effect, we hypothesize that (i) inositol exported from the vacuole via INT1 probably plays a different role than inositol synthesized via MIPS pathway in the cytosol and that (ii) a local INT1-mediated inositol gradient at the vacuolar membrane might be involved in regulatory processes in dependency of a certain sucrose concentration. The link between sucrose and inositol could not be assigned to a defined mechanism by our study, but it seems very likely that a certain set of (regulatory) proteins is only synthesized and/or active under sucrose conditions, going in line with the fact that a multitude of genes are differentially expressed by addition of sugars (Aghdasi et al., 2008; Lee et al., 2012; Lilley et al., 2012; Rottmann et al., 2016).

## Data Availability

The raw data supporting the conclusions of this manuscript will be made available by the authors, without undue reservation, to any qualified researcher.

## Author Contributions

SS conceived and planned the experiments, with supporting ideas from IH. SMS, DK, and SS carried out all experiments except lipid analysis, which was performed by IH and cell wall analysis, which was performed by GM. SS wrote the manuscript with input from all authors.

## Conflict of Interest Statement

The authors declare that the research was conducted in the absence of any commercial or financial relationships that could be construed as a potential conflict of interest.
