# Supplementary material for: The Tonoplastic Inositol Transporter INT1 From Arabidopsis thaliana Impacts Cell Elongation in a Sucrose-Dependent Way
Source: Front Plant Sci. 2018 Nov 16;9:1657. doi: 10.3389/fpls.2018.01657 (PMC6250803; doi:10.3389/fpls.2018.01657)
Supplement: Supplementary file 1 [file Table_1.pdf]

Table S1: Oligonucleotides

Oligonucleotides used for qPCR	
AtIMPL1cs551f	TCG GTG TTC TGT ATC GTG GA
AtIMPL1cs650r	CCT GCA GTT GCA GAA AAG GT
AtMIPS1cs1333f	CAG TTC AAA TCC GAG GGA GA
AtMIPS1cs1421r	GGT ACA AGT GGT GCC TTG GT
AtMIOX2cs699f	GGC TAA GAA GAA TGG CAC GA
AtMIOX2cs780r	TGC CTT GTG CAA TGG ATA AA
AtPI4Kαcs3466f	CGA GAA ACT TGT TCC CAA GC
AtPI4Kαcs3571r	ACC AAC AAA GGA GAC GCA GT
AtPI4Kβ1cs1486f	CGC AAG TTG TTC AGA GAT CG
AtPI4Kβ1cs1590r	ATC ATC CCG CTG TTT AGG TG
AtPI4Kβ2cs2312f	GTC GAA AGG AAC ATC GTC GT
AtPI4Kβ2cs2415r	AGC CCC TTT GAG AGG AAG AC
AtPIP5K2cs1193f	CGG AGG GTT CTA AGA CCA CA
AtPIP5K2cs1294r	CCA CCG TGA ATA GCT CCC TA
AtPIP5K9cs2107f	AGA TTA CGA GCA TCC GCT GT
AtPIP5K9cs2220r	CGG TAT CAG CTC TGC TCT TG
AtPIS1cs301f	TTC CTC TCA TTG CTG GCT TT
AtPIS1cs391r	TCA CAT CCT TAT GGC TGC TCT
AtPIS2cs86f	TTG CCT TCT CTG TGT GCT TC
AtPIS2cs186r	AAA TTT ACG AGC GCA CCA TC
AtPLC2cs451f	ATATGGCCCAATTCCAACAA
AtPLC2cs560r	TCAAAGGCATGTGCTCTGAT
AtSAL1cs207f	ACT CGC TGC TCG TCT CTG TC
AtSAL1cs296r	GCA ACG GTC ACT GGA CTT TT
AtVTC4.1cs391f	CTA TTC ACC GGT GTC CAA GG
AtVTC4.1cs478r	GCA AAG CGG TTA AAA GTT CG
UBQ10-qPCR-fwd	GAT GGT CGT ACT TTG GCG GAT TAC
UBQ10-qPCR-rev	AGA CGC AAC ACC AAG TGA AGG G

Oligonucleotides used for cloning	
AtINT1-5’SbfI	TAC CTG CAG GAT GAC ATT GAC GAT C
AtINT1-3’-stop-XbaI	ATT CTA GAT TAA GAT TGA GAT CCC TGC TCG A
e35S-5‘-HindIII	ATA AGC TTG CAC ATG GTG GAG C
e35S-3‘-SbfI	ATC CTG CAG GAT CCC CGG GTA CC
InsP 5-ptase-5‘-SbfI	TAC CTG CAG GAT GGC GGG GAA GGC
InsP 5-ptase-3‘-XbaI	ATT CTA GAT TAC TGC ACG ACA CAA CAC
