# Supplementary figures and images for: The Tonoplastic Inositol Transporter INT1 From Arabidopsis thaliana Impacts Cell Elongation in a Sucrose-Dependent Way
Source: Front Plant Sci. 2018 Nov 16;9:1657. doi: 10.3389/fpls.2018.01657 (PMC6250803; doi:10.3389/fpls.2018.01657)

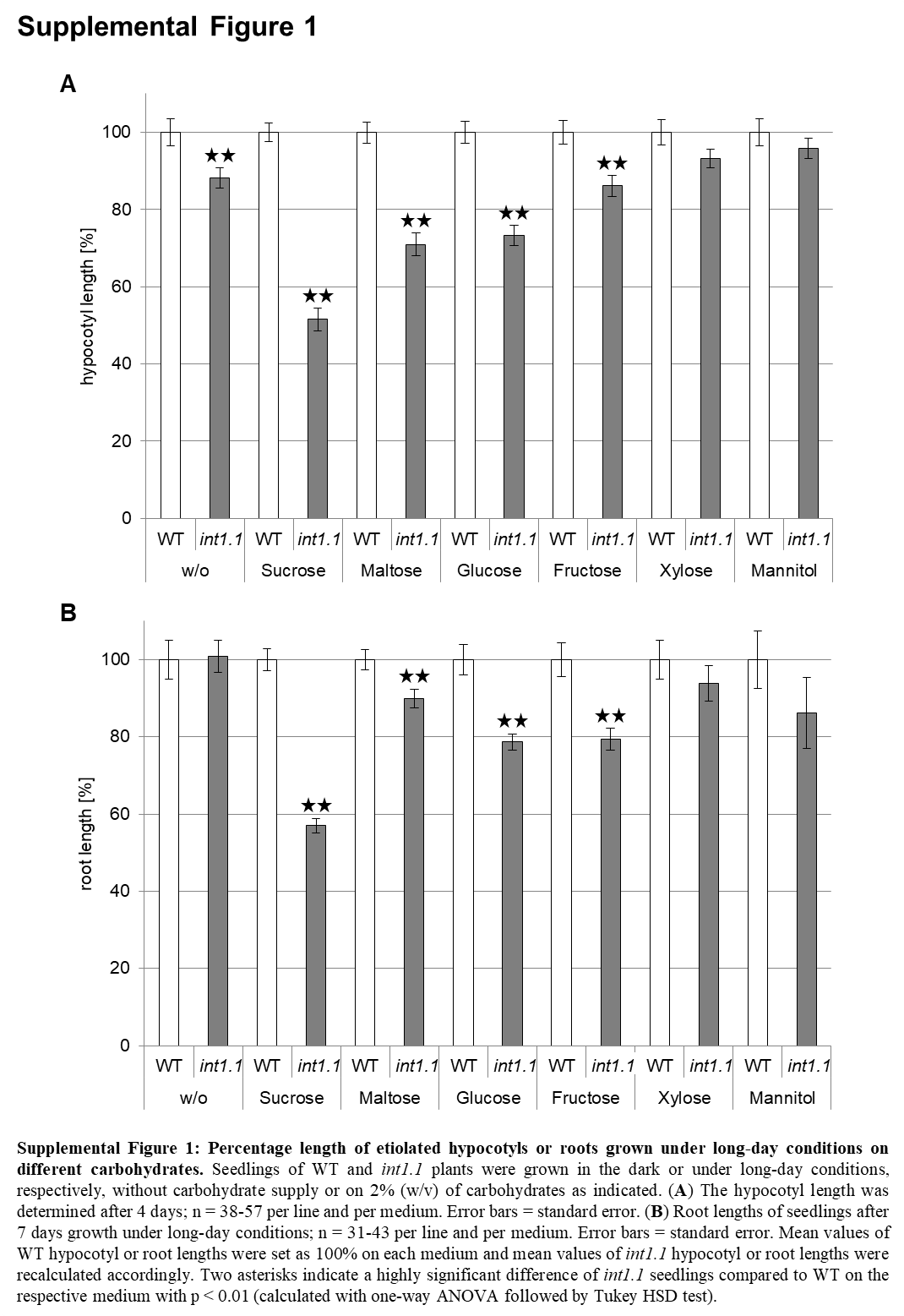

Supplement: Supplementary file 2 [file Image_1.tif]

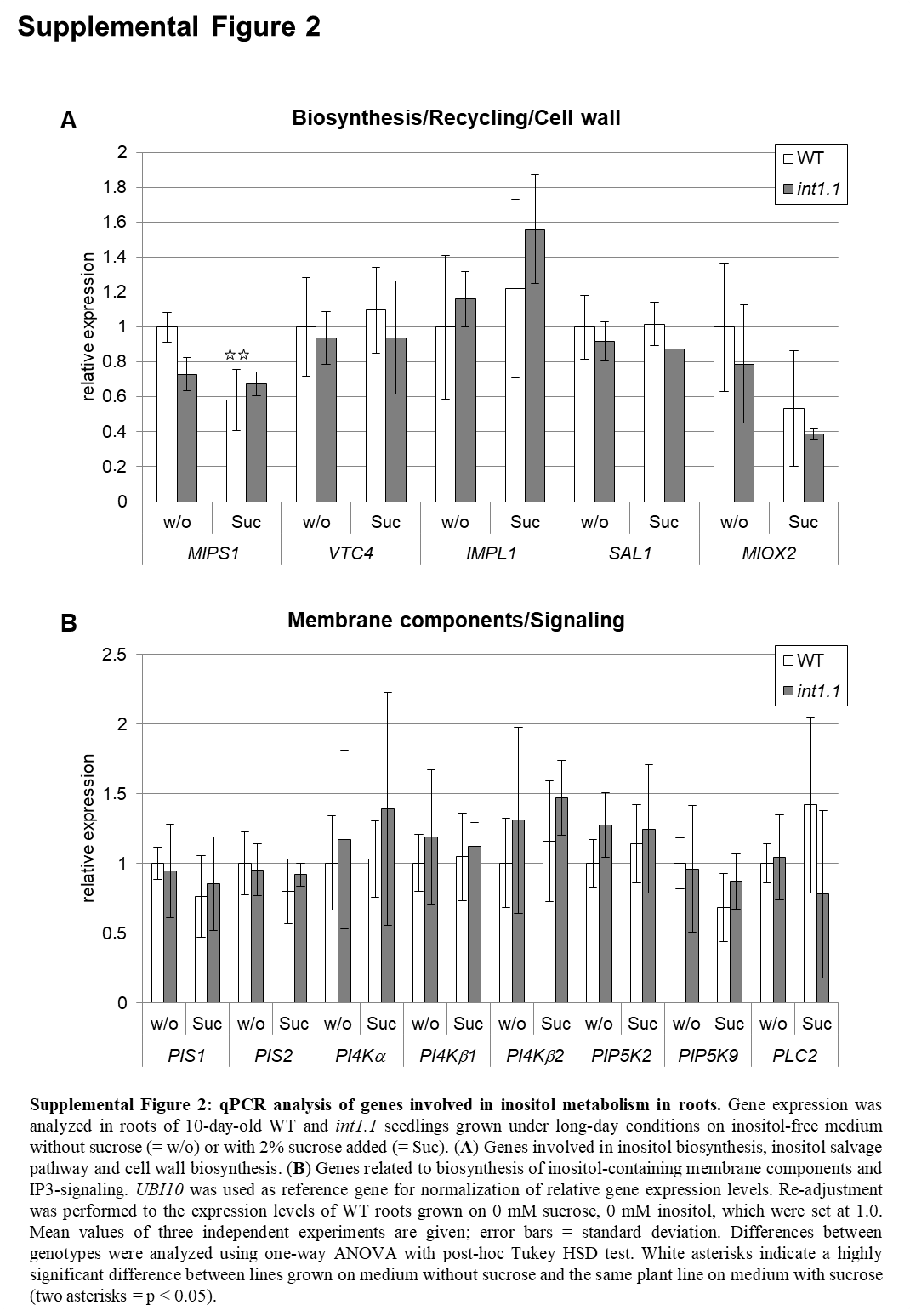

Supplement: Supplementary file 3 [file Image_2.tif]

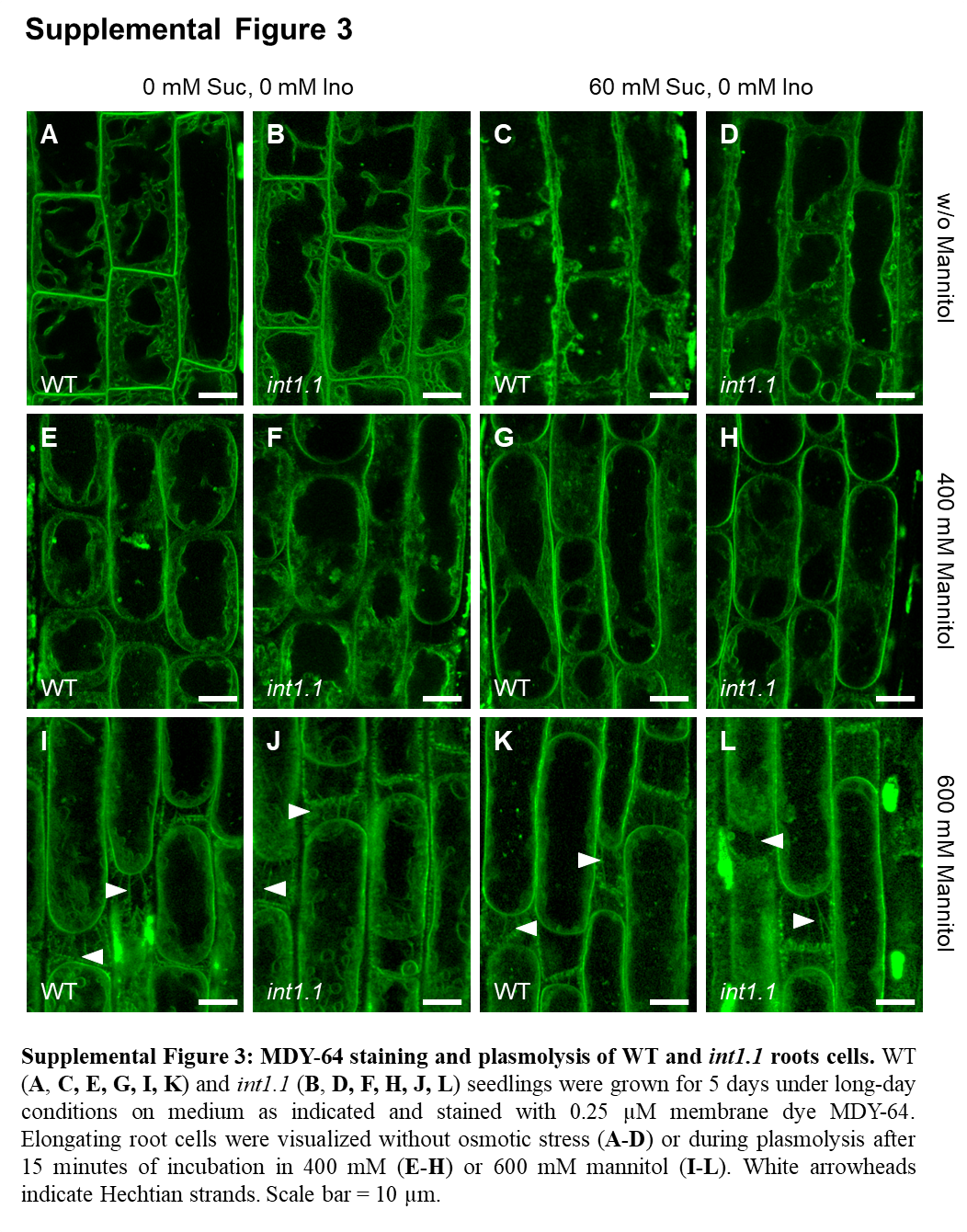

Supplement: Supplementary file 4 [file Image_3.tif]

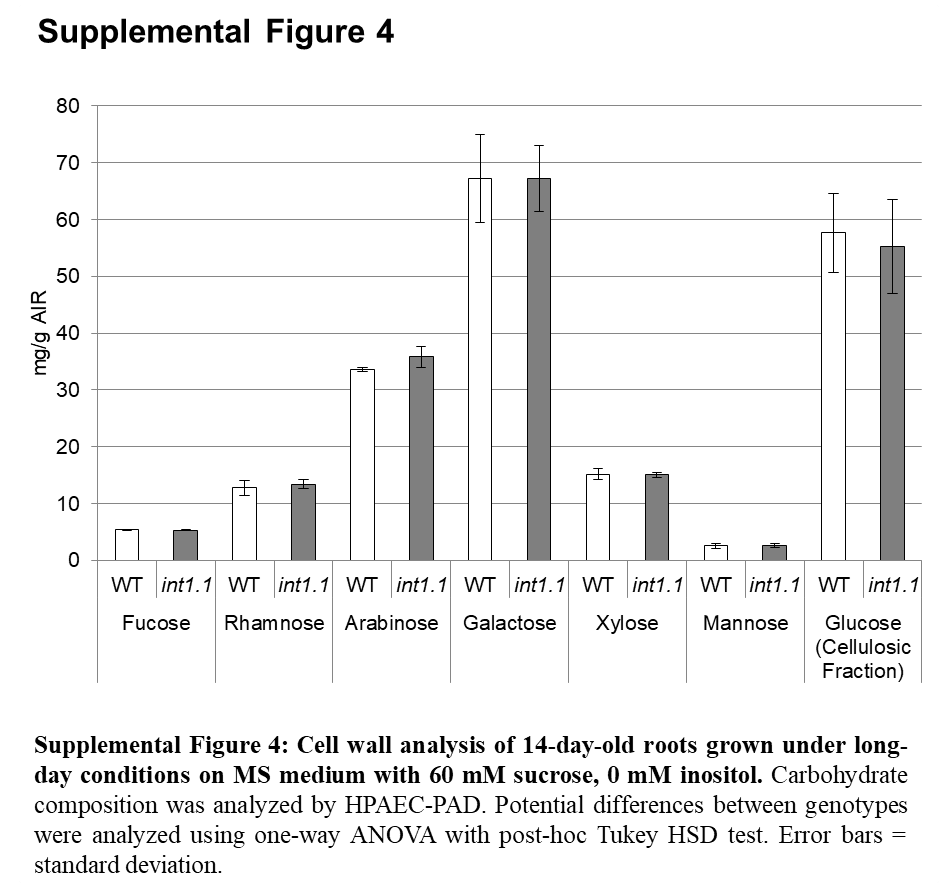

Supplement: Supplementary file 5 [file Image_4.tif]

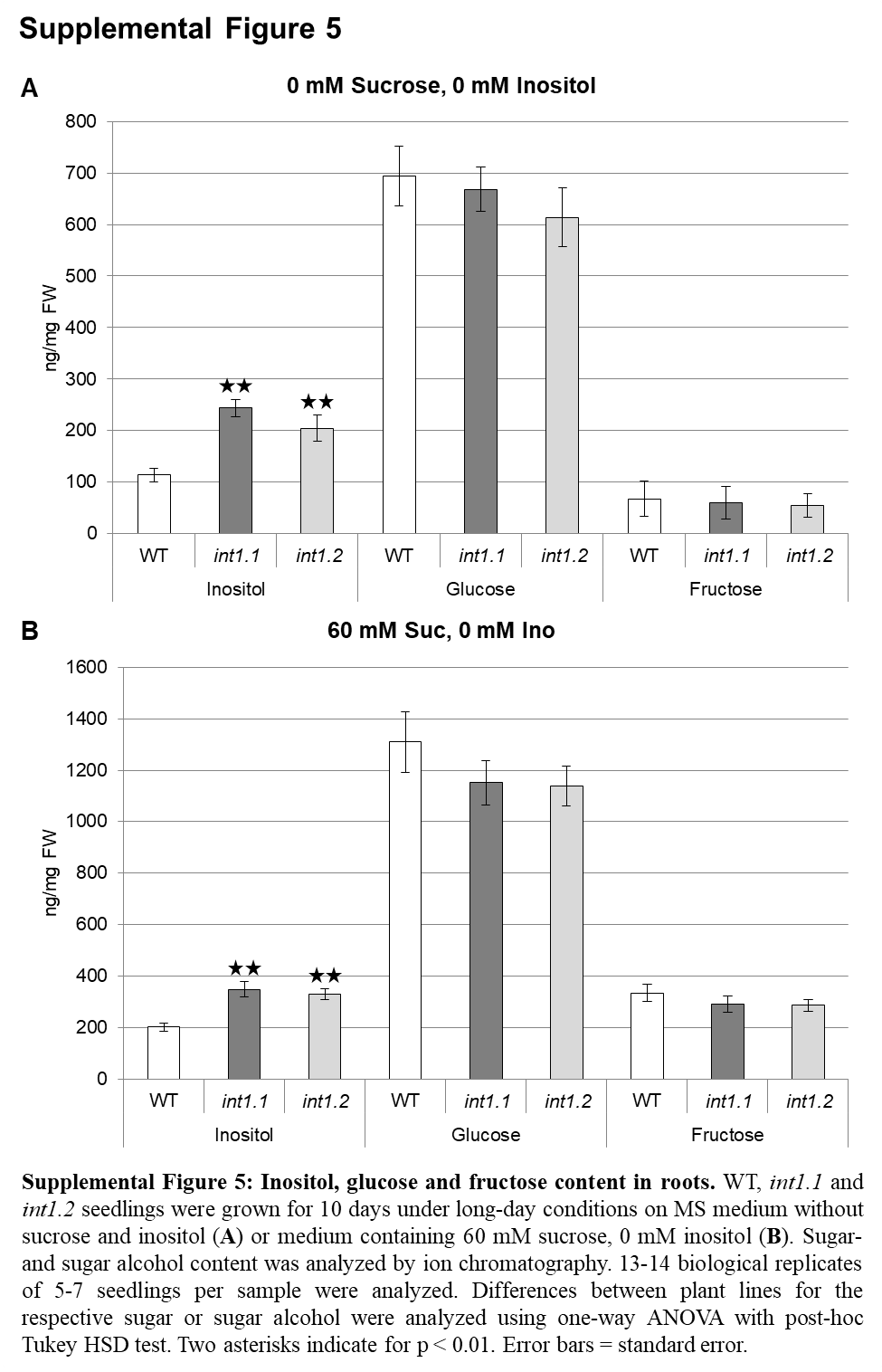

Supplement: Supplementary file 6 [file Image_5.tif]

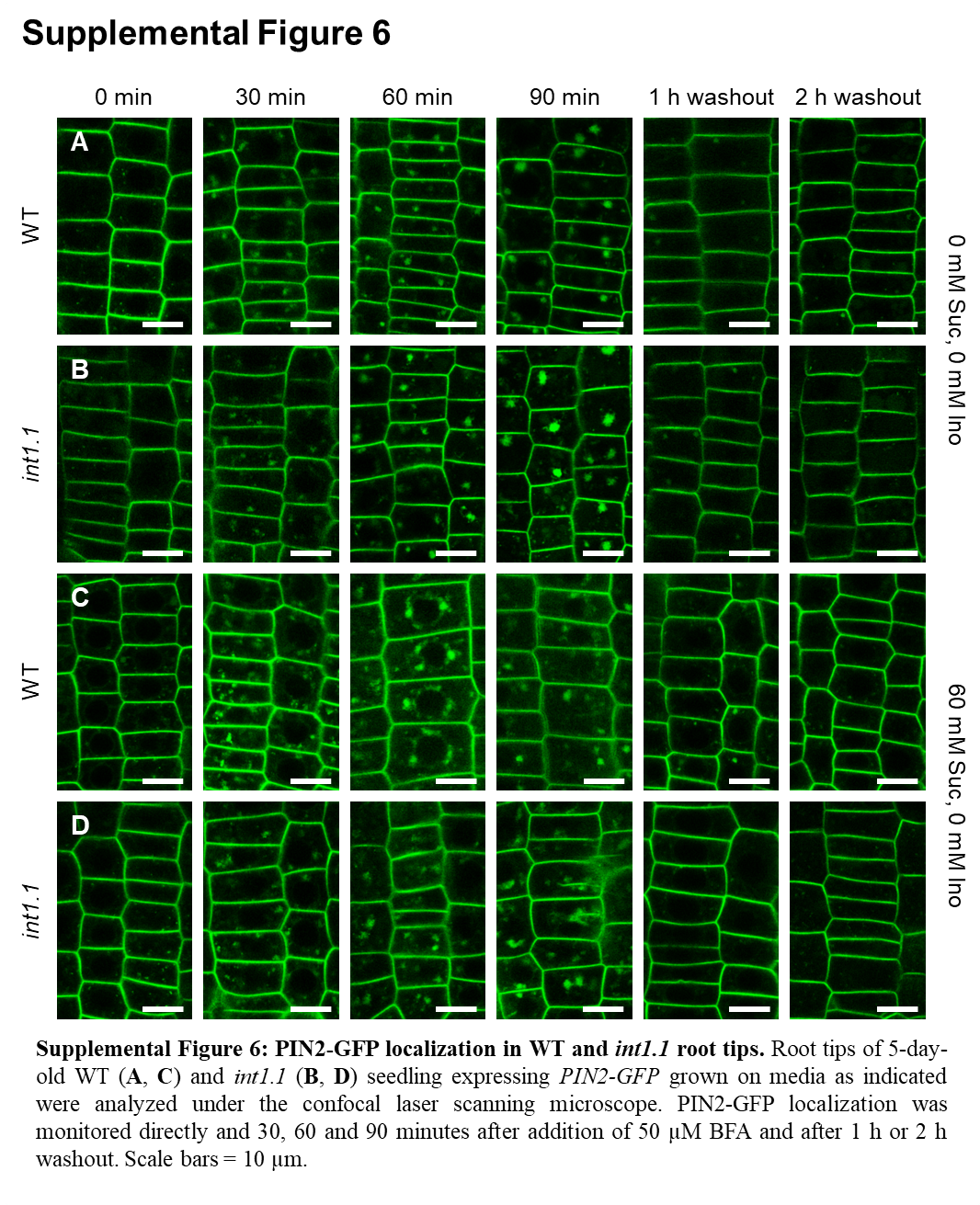

Supplement: Supplementary file 7 [file Image_6.tif]

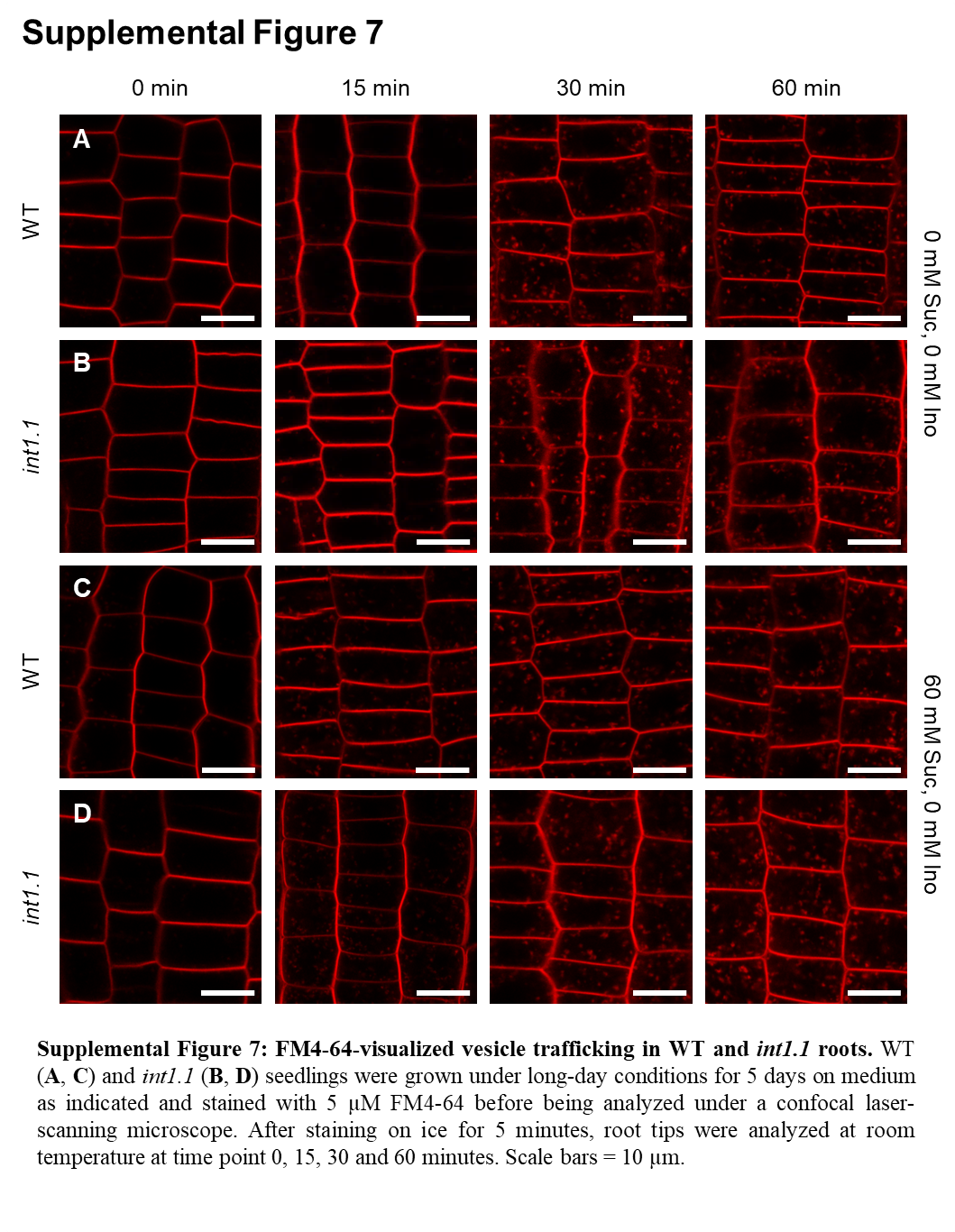

Supplement: Supplementary file 8 [file Image_7.tif]

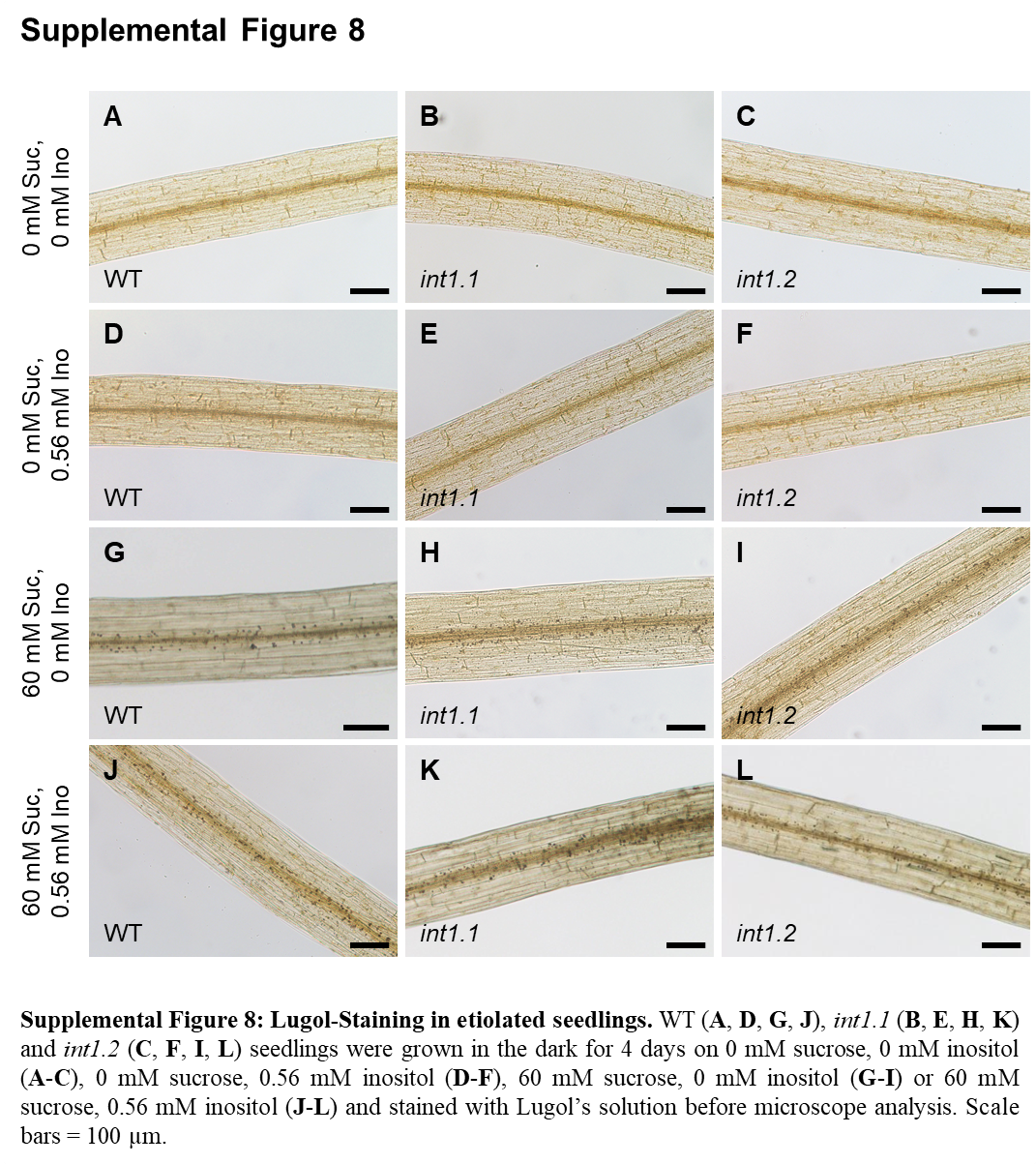

Supplement: Supplementary file 9 [file Image_8.tif]

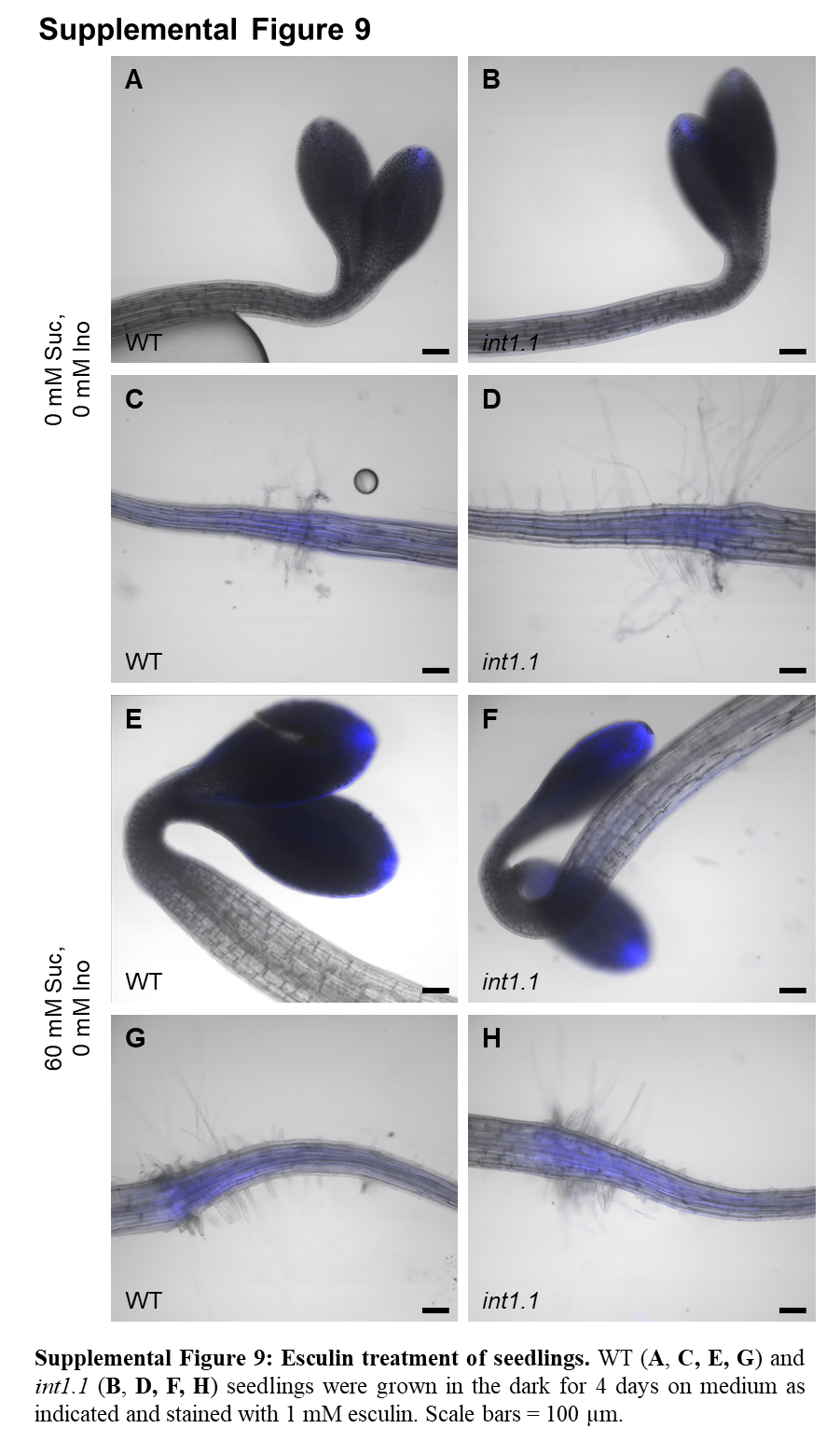

Supplement: Supplementary file 10 [file Image_9.tif]
